# Gene Expression and Protein Abundance of Hepatic Drug Metabolizing Enzymes in Liver Pathology

**DOI:** 10.3390/pharmaceutics13091334

**Published:** 2021-08-25

**Authors:** Marek Drozdzik, Joanna Lapczuk-Romanska, Christoph Wenzel, Sylwia Szelag-Pieniek, Mariola Post, Łukasz Skalski, Mateusz Kurzawski, Stefan Oswald

**Affiliations:** 1 Department of Experimental and Clinical Pharmacology, Pomeranian Medical University, Powstancow Wlkp. 72 Str., 70-111 Szczecin, Poland; joanna.lapczuk@pum.edu.pl (J.L.-R.); sylwia.szelag@pum.edu.pl (S.S.-P.); lukasz.skalski@pum.edu.pl (Ł.S.); mkurz@pum.edu.pl (M.K.); 2 Department of Pharmacology, Center of Drug Absorption and Transport, University Medicine Greifswald, 17489 Greifswald, Germany; Christoph.Wenzel@med.uni-greifswald.de; 3 Department of General and Transplantation Surgery, County Hospital, 70-891 Szczecin, Poland; mariolapost@wp.pl; 4 Institute of Pharmacology and Toxicology, Rostock University Medical Center, 18051 Rostock, Germany; stefan.oswald@med.uni-rostock.de

**Keywords:** hepatic pathology, liver, drug metabolizing enzymes

## Abstract

Hepatic drug metabolizing enzymes (DMEs) markedly affect drug pharmacokinetics. Because liver diseases may alter enzymatic function and in turn drug handling and clinical efficacy, we investigated DMEs expression in dependence on liver pathology and liver failure state. In 5 liver pathologies (hepatitis C, alcoholic liver disease, autoimmune hepatitis, primary biliary cholangitis and primary sclerosing cholangitis) and for the first time stratified according to the Child–Pugh score, 10 CYPs (CYP1A1, CYP1A2, CYP2B6, CYP2C8, CYP2C9, CYP2C19, CYP2D6, CYP2E1, CYP3A4 and CYP3A5) and 4 UGTs (UGT1A1, UGT1A3, UGT2B7 and UGT2B) enzymes were quantified for protein abundance (LC-MS/MS) and gene expression (qRT-PCR). CYP2E1 was the most vulnerable enzyme, and its protein levels were significantly reduced just in Child–Pugh class A livers. The protein abundance of CYP1A1, CYP2B6, CYP2C19, CYP2D6 as well as UGT1A1, UGT1A3 and UGT2B15 was relatively stable in the course of progression of liver function deterioration. Alcoholic liver disease and primary biliary cholangitis were involved in the most prominent changes in the protein abundances, with downregulation of 6 (CYP1A2, CYP2C8, CYP2D6, CYP2E1, CYP3A4, UGT2B7) and 5 (CYP1A1, CYP2B6, CYP2C8, CYP2E1, CYP3A4) significantly downregulated enzymes, respectively. The results of the study demonstrate that DMEs protein abundance is affected both by the type of liver pathology as well as functional state of the organ.

## 1. Introduction

Drug metabolizing enzymes (DMEs) expressed in the liver play a very important role in drug biotransformation, and together with enzymes in the gastrointestinal tract, are major bioavailability determinants of orally administered pharmaceuticals and drug–drug interactions. The available pharmacokinetic information suggests deficient function of the enzymes in liver dysfunction states, e.g., increased bioavailability of orally administered caffeine (CYP1A2 substrate), S-mephenytoin (CYP2C19 substrate), debrisoquine (CYP2D6 substrate), chlorzoxazone (CYP2E1 substrate) and midazolam (CYP3A4 substrate) or not affected pharmacokinetics of debrisoquine [1,2,3]. However, other factors observed in cirrhotic patients may also contribute to the altered drug pharmacokinetics, e.g., reductions in portal and renal blood flow, glomerular filtration rate, serum albumin concentration or hematocrit value, altered membrane transporter expression in the liver and other tissues and the decrease in the remaining functional liver mass [4,5,6].

The available findings demonstrated that about 30% of patients with advanced liver failure suffered from adverse drug reactions and that almost 80% of those reactions were possibly preventable because inadequate dosages or contraindicated medicines were used [7]. Therefore, European Medicines Agency (EMA) and Food and Drug Administration (FDA) regulations recommend pharmacokinetic studies in patients with impaired hepatic function, when hepatic impairment is likely to significantly alter the pharmacokinetics (especially metabolism and biliary excretion) of the drug and/or its active metabolites (i.e., hepatic metabolism and/or excretion accounting for a substantial portion (>20 percent of the absorbed drug) of the elimination of a parent drug or active metabolite]. Dose adjustment may be required for such patients, taking into account the PK/PD relationship resulting in increased rates of side effects or drug-related toxicity [8,9]. However, ethical concerns virtually preclude methodologically rigorous pharmacokinetic or drug–drug interaction studies, especially for medicines of non-therapeutic value. This gap of knowledge may be to some extent compensated by proteomic studies, which provide quantitative data on hepatic DMEs in pathological states of the organ. The information about DMEs abundance in liver dysfunction states is of clinical relevance, as it enables stratification of potential risks derived from altered pharmacokinetics and pharmacodynamics of administered drugs and prediction of oral drug bioavailability and drug–drug interactions (DDI) using physiologically-based pharmacokinetic (PBPK) modeling and simulation.

The published information about protein abundance of DMEs in liver pathologies is very scarce. Several studies reported gene expression analysis. However, mRNA data may not be necessarily correlated to the encoded proteins [10,11]. The so far available protein-based data are limited to alcoholic liver disease and hepatitis C cirrhosis, as measured by liquid chromatography coupled with tandem mass spectrometry (LC-MS/MS) or in cholestatic cirrhosis (assayed by Western blot) [12,13,14]. Except for Guengerich et al., those reports did not stratify patients according to the functional state of the organ, i.e., the established Child–Pugh score [13]. Moreover, protein quantification by Western blotting regularly does not allow absolute protein quantification and is associated with several analytical issues such as unspecific binding of the antibodies, a narrow analytical range, which is limited to only a few proteins, a low samples throughput and a lack of reliable reproducibility [15].

In order to overcome the aforementioned limitations, a validated targeted proteomics method and quantitative RT-PCR were used to determine, respectively, protein abundance and mRNA levels of hepatic DMEs in a wide range of liver pathological states, i.e., hepatic virus C (HCV)-induced liver damage, alcoholic liver disease (ALD), autoimmune hepatitis (AIH) and cholestatic diseases, such as primary biliary cholangitis (PBC) and primary sclerosing cholangitis (PSC), in patients stratified according to the degree of hepatic insufficiency (based on the Child–Pugh score). The following enzymes were analyzed: CYPs (CYP1A1, CYP1A2, CYP2B6, CYP2C8, CYP2C9, CYP2C19, CYP2D6, CYP2E1, CYP3A4 and CYP3A5) and UGTs (UGT1A1, UGT1A3, UGT2B7 and UGT2B15) enzymes. The data from the study could be applied to develop more accurate PBPK-based prediction models for pathology states of the liver. The current study is complementary (includes the same set of the studied samples) to the previously published information about hepatic drug transporters in different forms of liver failure [5].

## 2. Materials and Methods

### 2.1. Liver Samples

The control samples were harvested from metastatic livers, from a site at least 5 cm distant of the tumor site. The tissues were collected from Caucasian patients, aged 63 ± 10 years, 11 males and 9 females, diagnosed with metastatic colon cancer. The collected tissues did not show any pathological signs as confirmed by histological examination. The hepatitis C (HCV), primary biliary cholangitis (PBC), primary sclerosing cholangitis (PSC), alcoholic liver disease (ALD) and autoimmune hepatitis (AIH) (diagnosed according to the standard clinical criteria) liver parenchymal tissue samples were dissected from patients requiring liver transplantation. The liver tissue specimens were harvested during elective liver transplantation from the organ, immediately after excision. The stage of liver dysfunction was classified according to the Child–Pugh score. Characteristics of the subjects are presented in Table S1. The whole medication information is available for the control samples, i.e., one patient took bisoprolol, furosemide, and tamsulosin (hypertension, prostate hypertrophy), one was treated with bepridil (hypertension) and another one was medicated with amlodipine (hypertension). None of these drugs is known to be a potent regulator of CYP or UGT enzymes. The liver pathology samples were collected in the years 2007–2018, and treatment standards for the liver diseases were modified, and are also specific liver pathology and functional state dependent, therefore due to limited number of samples for a given liver pathology and functional state medication analysis is not reliable (which is a limitation of these samples). We were only able to select samples without co-existing co-morbidities. Tissue biopsies were taken from livers (control and pathological) under standard general anesthesia (propofol, sevoflurane, rocuronium, fentanyl, or dipyrone) not later than 15 min after blood flow arrest or directly after liver resection in metastatic controls. The liver samples were immediately snap frozen in liquid nitrogen for protein analysis or immersed in RNAlater (Applied Biosystems, Darmstadt, Germany) for RNA analysis, and then stored at −80 °C. The study protocol was approved by Bioethics Committee of the Pomeranian Medical University (approval number BN-001/11/07). Informed consent was obtained from all subjects involved in the study.

### 2.2. mRNA Isolation and Quantitative Real-Time RT-PCR

Total RNA was isolated from 25 mg of each tissue sample using Direct-zol RNA MiniPrep kit (Zymo Research, Irvine, CA, USA). RNA concentration and purity was assessed using DS-11 FX spectrophotometer (Denovix, Wilmington, DE, USA). cDNA was prepared using SuperScript^®^ VILO™ cDNA Synthesis Kit (Thermo Fisher Scientific, Waltham, MA, USA), with 500 ng of total RNA for 20 µL of reaction volume according to the manufacturer’s procedure. The gene expression levels were examined in duplicate using TaqMan Fast Advanced Master Mix and pre-validated TaqMan assays: CYP1A1 (manufacturer’s Assay ID: Hs00153120_m1), CYP1A2 (Hs00167927_m1), CYP2B6 (Hs03044631_m1), CYP2C8 (Hs02383390_s1), CYP2C9 (Hs02383631_s1), CYP2C19 (Hs00426380_m1), CYP2D6 (Hs00164385_m1), CYP2E1 (Hs00559367_m1), CYP3A4 (Hs00604506_m1), CYP3A5 (Hs01070905_m1), UGT1A1 (Hs02511055_s1), UGT1A3 (Hs04194492_g1), UGT2B7 (Hs00426592_m1), UGT2B15 (Hs00870076_s1) in ViiA 7 Real-Time PCR System (Life Technologies, Carlsbad, CA, USA). Threshold values for each gene were set manually and mean CT (cycles of threshold) values were recorded. Relative mRNA expression was calculated by the 2-ΔCt method- normalized to the mean expression value obtained for the housekeeping genes: GAPDH (Hs99999905_m1), HMBS (Hs00609297_m1), PPIA (Hs04194521_s1), RPLP0 (Hs99999902_m1), RPS9 (Hs02339424_g)—presented in the figures and by 2-ΔΔCt method- additionally normalized to the mean value for the control group—presented in the table (Supplementary Table S2).

### 2.3. Protein Quantification by LC−MC/MS

The tissues placed in liquid nitrogen were mechanically disrupted in a stainless steel mortar system. Approximately 40 mg tissue powder of each sample was lysed with 1 mL of 0.2% SDS and 5 mM EDTA containing 5 µL/mL Protease Inhibitor Cocktail Set III (Merck, Darmstadt, Germany) for 30 min at 4 °C on a platform shaker with 40 rpm (Polymax 1040, Heidolph, Schwabach, Germany). Total protein content of the whole tissue lysates was determined by bicinchoninic acid assay (Thermo Fisher Scientific, Waltham, MA, USA) and 100 µg of each sample was processed using filter aided sample preparation (FASP) based on the previously published protocol [16]. Protein abundances of nine CYP (CYP1A2, CYP2B6, CYP2C8, CYP2C9, CYP2C19, CYP2D6, CYP2E1, CYP3A4, CYP3A5) and four UGT (UGT1A1, UGT1A3, UGT2B7 and UGT2B15) enzymes were measured by mass spectrometry-based targeted proteomics using a validated LC−MS/MS method as previously described [16,17]. The analytical variability during sample analysis was below 20% (accuracy). With the exception of UGT1A3, two proteospecific peptides were used for analysis. One peptide was used for quantification, whereas the other served as qualifier for the presence of the specific protein. For all peptides and their isotope-labeled internal standard peptides, three mass transitions were used, respectively. The calculated protein (per mg liver tissue) values represent the mean of at least 2–3 mass transitions/peptide.

### 2.4. Genotyping

Genomic DNA was extracted from tissue samples using Tissue DNA Purification Kit (EURx, Gdansk, Poland), subsequently standardized to a uniform concentration (20 ng/μL) and stored in −20 °C. All samples were genotyped for common lack-of-function variants affecting protein concentrations (i.e., stop-codons, frameshifts and splicing defects, using ViiA7 Fast Real-Time PCR System and pre-validated TaqMan assays (Life Technologies, Carlsbad, CA, USA). Following variants were evaluated: CYP2C19*2 (rs4244285, Assay ID: C__25986767_70), CYP2D6*3 (rs35742686, C__32407232_50), CYP2D6*4: rs3892097, C__27102431_D0), and CYP3A5*3 (rs776746, C__26201809_30). Additionally, CYP2D6 gene deletion (CYP2D6*5) was evaluated using qPCR method with TaqMan probes for CYP2D6 (Hs00010001_cn) and the reference RPPH1 gene.

### 2.5. Statistical Analysis

All mRNA and protein level data: means ± standard deviation, coefficient of variation %, median as well as minimum and maximum values are given in Tables S2 and S3 of Supplementary Information. The final protein amounts were normalized to the digested tissue amount. All samples were used for calculations, and those with undetectable protein concentration (LLOQ, lower or equal to 0.1 nmol/L) were replaced with zero. Differences between study groups were evaluated using the nonparametric Kruskal–Wallis test for multiple comparisons with post hoc Dunn’s test with Bonferroni correction, and correlations the Spearman rank (rs) test. *p* values of <0.05 were considered as significant. All statistical calculations were performed using Statistica 13.3 Software Package (TIBCO Software Inc., Palo Alto, CA, USA).

## 3. Results

### 3.1. Hepatic Drug Metabolizing Enzymes Abundance According to Liver Functional State (The Child–Pugh Score)

**Figure 1:**
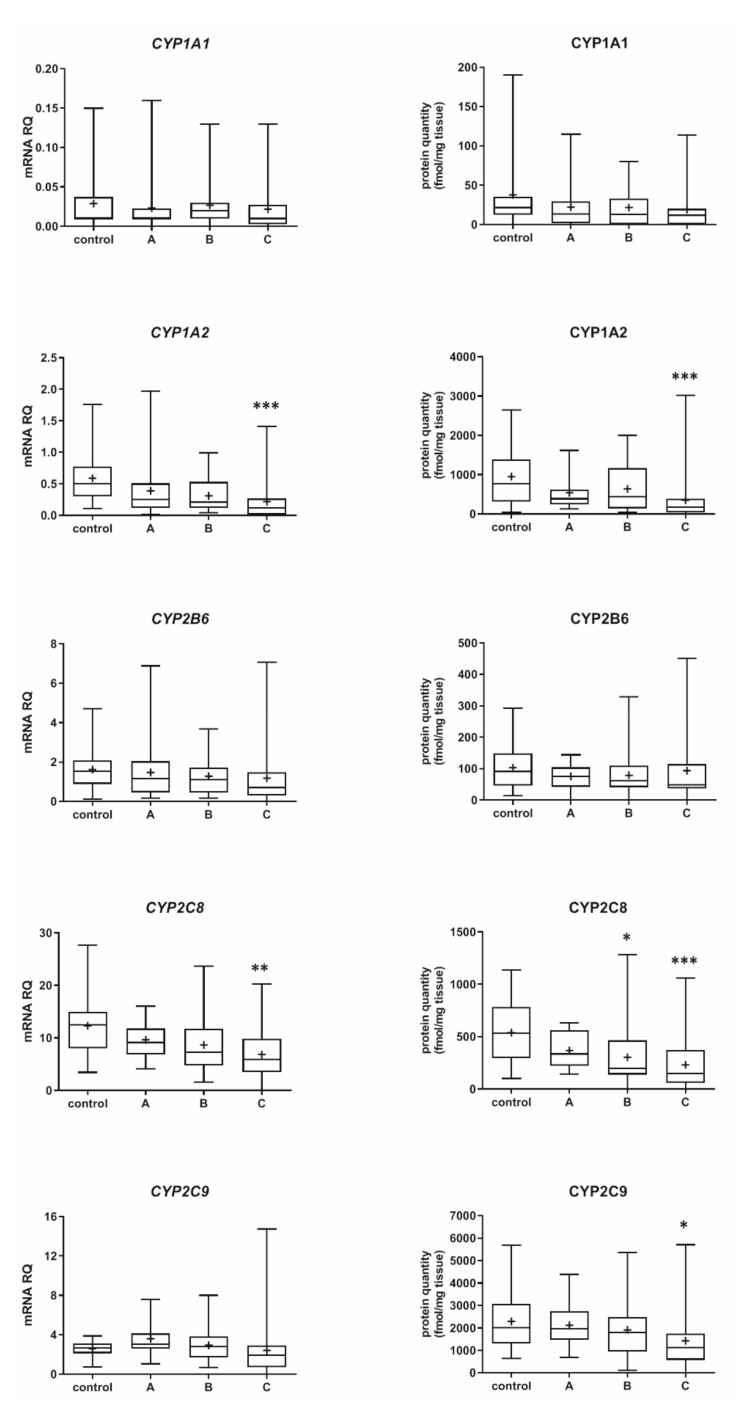

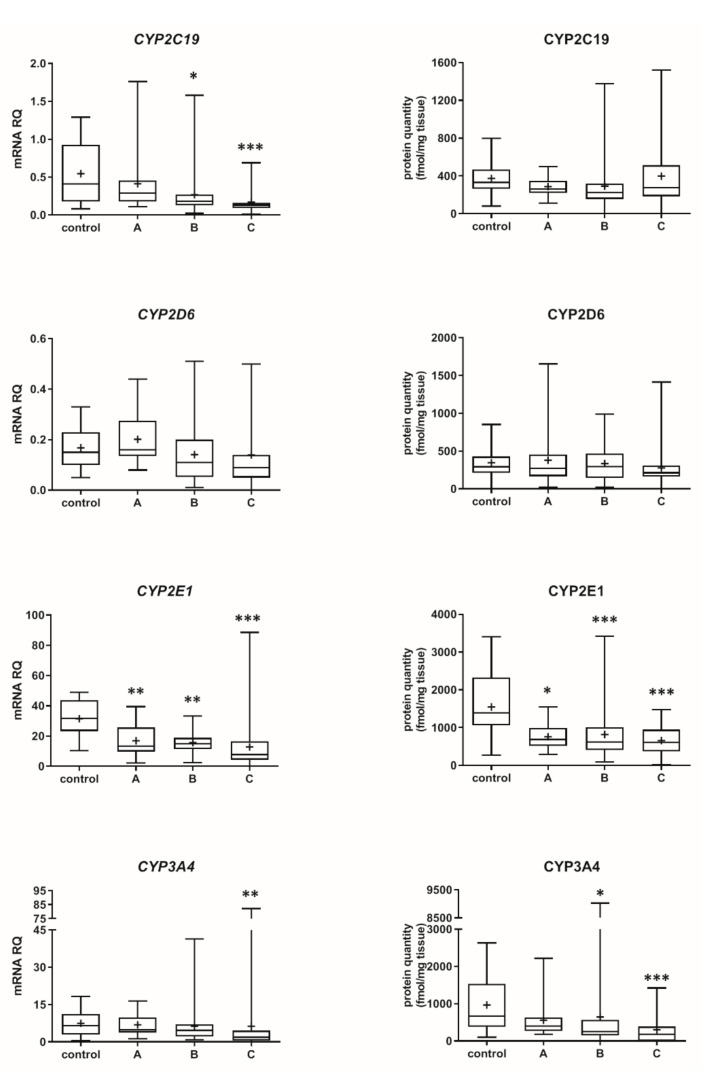
(also see page up) Gene expression (**left**) and protein abundance (**right**) of CYPs in hepatic tissues stratified according to the Child–Pugh score into stages: A (*n* = 18), B (*n* = 31) and C (*n* = 28). The data are represented as box-plots of the median (horizontal line), 75th (top of box), and 25th (bottom of box) quartiles, the smallest and largest values (whiskers) and mean (+) are shown. mRNA levels of the analyzed genes were expressed as relative amounts to the mean of five housekeeping genes (GAPDH, HMBS, PPIA, RPLP0, RPS9). Statistically significant differences: * *p* < 0.05, ** *p* < 0.01, *** *p* < 0.001 (Kruskal–Wallis test post hoc Dunn’s test with Bonferroni correction) in comparison to the controls.

**Figure 2:**
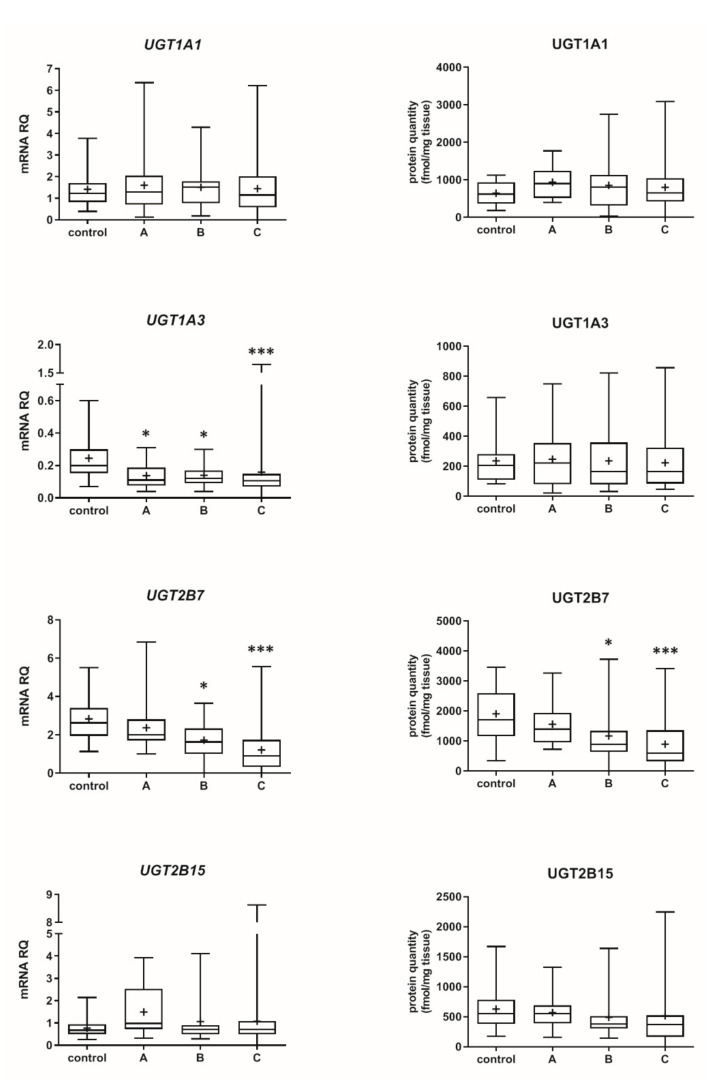
Gene expression (**left**) and protein abundance (**right**) of UGTs in hepatic tissues stratified according to the Child–Pugh score into stages: A (*n* = 18), B (*n* = 31) and C (*n* = 28). The data are represented as box-plots of the median (horizontal line), 75th (top of box), and 25th (bottom of box) quartiles, the smallest and largest values (whiskers) and mean (+) are shown. mRNA levels of the analyzed genes were expressed as relative amounts to the mean of five housekeeping genes (GAPDH, HMBS, PPIA, RPLP0, RPS9). Statistically significant differences: * *p* < 0.05, *** *p* < 0.001 (Kruskal–Wallis test post hoc Dunn’s test with Bonferroni correction) in comparison to the controls.

The Child–Pugh class was associated with changes in the transcriptional expression and protein levels of both, CYP and UGT enzymes. Worsening of liver function was associated with a significant decrease in protein abundance (compared to control samples) of CYP1A2 (to 45% in Child–Pugh score C), CYP2C8 (to 45% in Child–Pugh score C), CYP2C9 (to 57% in Child–Pugh score C), CYP2E1 (to 89% in Child–Pugh score C) and CYP3A4 (to 45% in Child–Pugh score C) as well as UGT2B7 (to 57% in Child–Pugh score C). The protein levels of CYP1A1, CYP2B6, CYP2C19 and CYP2D6 as well as that of UGT1A1, UGT1A3 and UGT2B15 remained stable. However, in some of the enzymes a downregulation trend was observed (Figure 1 and Figure 2, Tables S2 and S3). 

**Figure 3:**
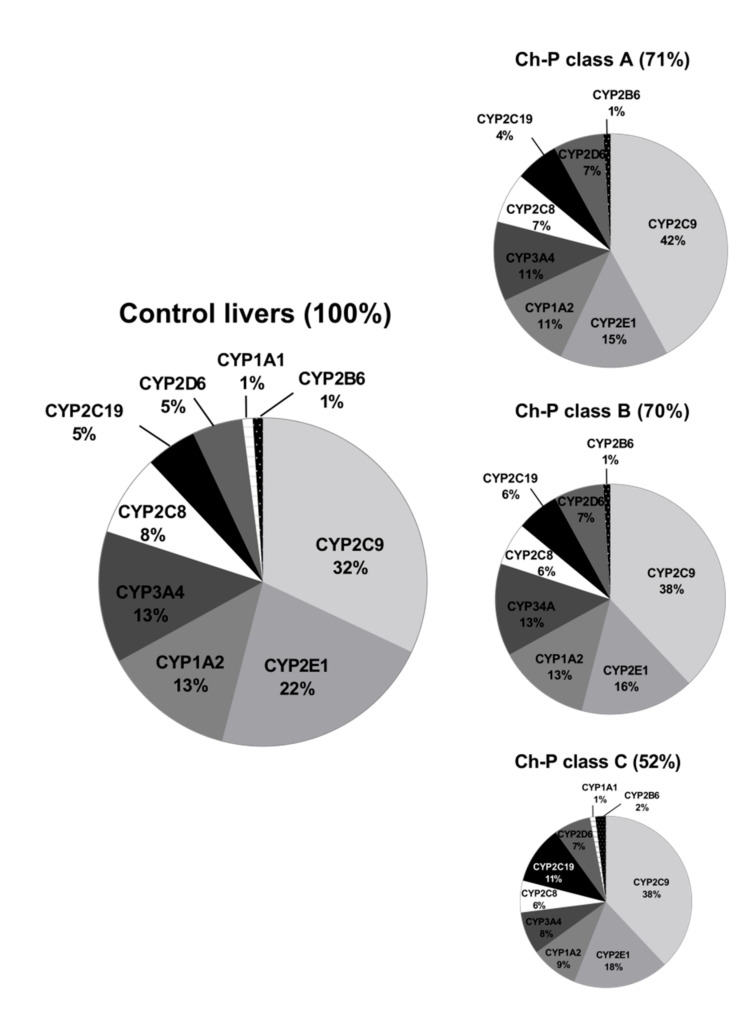
The pie chart of the individual enzyme proteins in livers stratified according to the Child–Pugh score (A, B, C class). The pie charts show the abundance of each enzymatic protein as a percentage of the sum of all enzyme proteins abundance. Percentages in brackets indicate a total enzyme protein abundance in comparison to the control livers (indicated as 100%).

The percentage contributions of all investigated CYP proteins stratified according to the different Child–Pugh scores are given in Figure 3. Although the protein amounts and rank order of the enzymes were markedly affected by the liver functional states, CYP2C9 and CYP2E1 showed the highest abundances, while CYP2B6 was only found in traces (~1–2%) in all liver pathologies (Figure 3, Table S3). Interestingly, CYP1A1 could be only detected in the control and the Child–Pugh score C livers at very low levels (1%). 

Among the studied UGTs, the most abundant isoenzyme was UGT2B7, and the least one was UGT1A3, irrespectively of the stage of liver dysfunction (Table S3). Details on differences in the DMEs mRNA and protein abundance levels between the studied liver pathologies are presented in Table S4. 

### 3.2. Hepatic Drug Metabolizing Enzymes in Different Forms of Liver Disease

**Figure 4:**
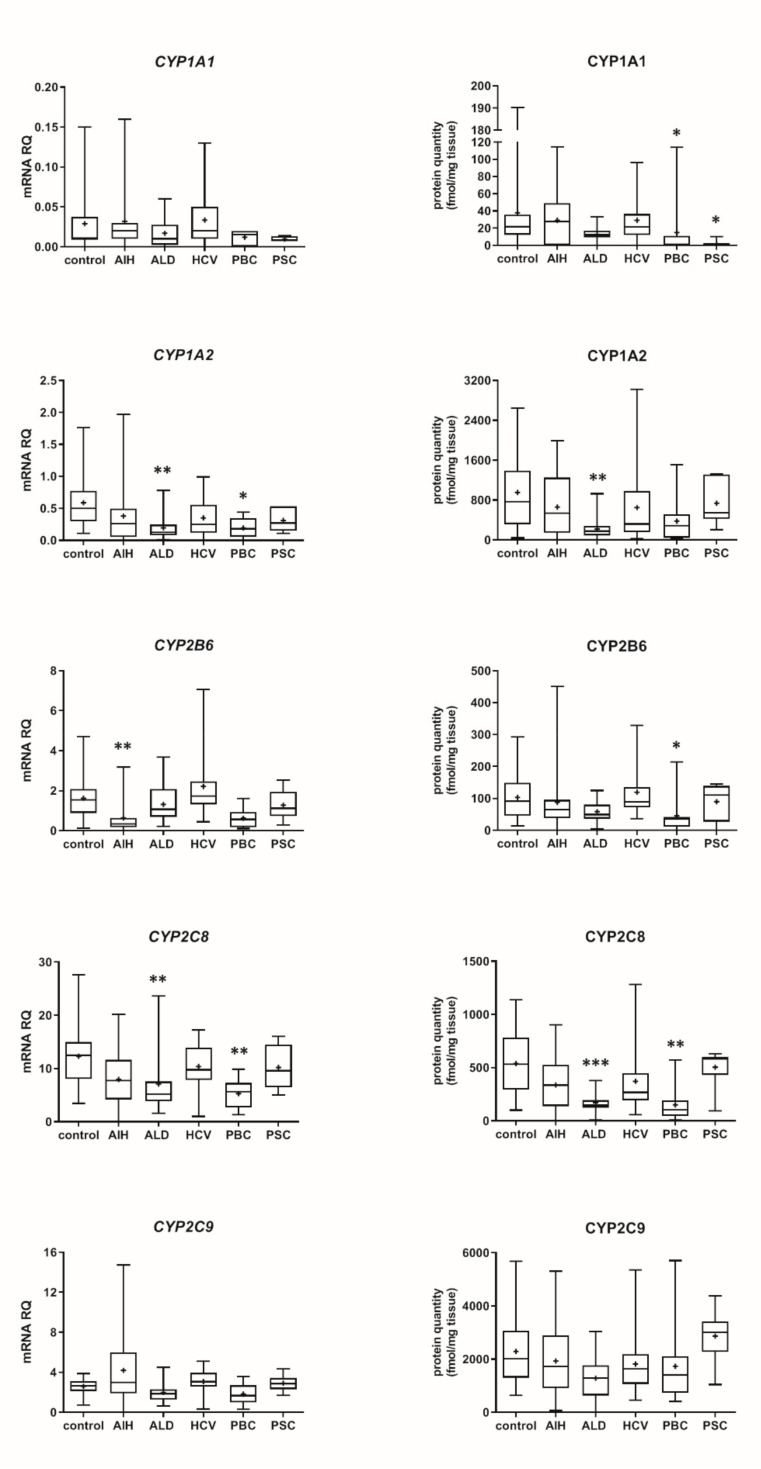

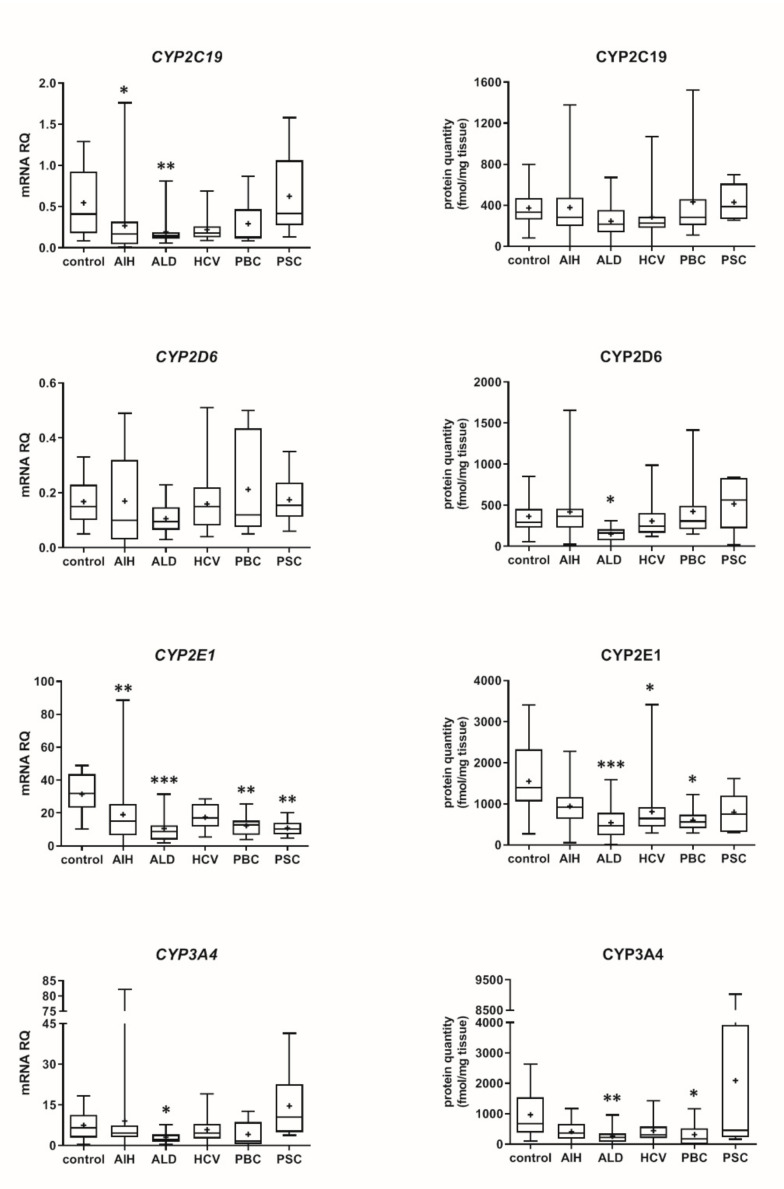
(also see page up) Gene expression (**left**) and protein abundance (**right**) of CYPs in hepatic tissues from hepatitis C (HCV, *n* = 21), primary biliary cholangitis (PBC, *n* = 10), primary sclerosing cholangitis (PSC, *n* = 6), alcoholic liver disease (ALD, *n* = 20) and autoimmune hepatitis (AIH, *n* = 20) patients and the controls (*n* = 20). The data are represented as box-plots of the median (horizontal line), 75th (top of box), and 25th (bottom of box) quartiles, the smallest and largest values (whiskers) and mean (+) are shown. mRNA levels of the analyzed genes were expressed as relative amounts to the mean of five housekeeping genes (*GAPDH*, *HMBS*, *PPIA*, *RPLP0*, *RPS9*). Statistically significant differences: * *p* < 0.05, ** *p* < 0.01, *** *p* < 0.001 (Kruskal–Wallis test post hoc Dunn’s test with Bonferroni correction) in comparison to the controls.

**Figure 5:**
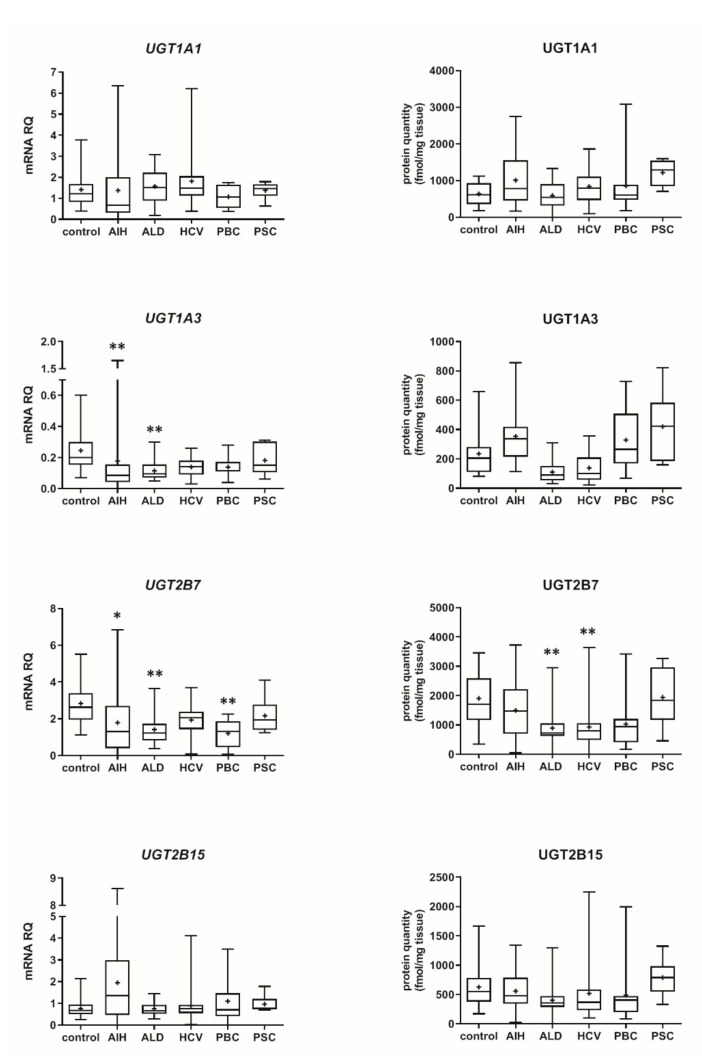
Gene expression (**left**) and protein abundance (**right**) of UGTs in hepatic tissues from hepatitis C (HCV, *n* = 21), primary biliary cholangitis (PBC, *n* = 10), primary sclerosing cholangitis (PSC, *n* = 6), alcoholic liver disease (ALD, *n* = 20) and autoimmune hepatitis (AIH, *n* = 20) patients and the controls (*n* = 20). The data are represented as box-plots of the median (horizontal line), 75th (top of box), and 25th (bottom of box) quartiles, the smallest and largest values (whiskers) and mean (+) are shown. mRNA levels of the analyzed genes were expressed as relative amounts to the mean of five housekeeping genes (GAPDH, HMBS, PPIA, RPLP0, RPS9). Statistically significant differences: * *p* < 0.05, ** *p* < 0.01 (Kruskal–Wallis test post hoc Dunn’s test with Bonferroni correction) in comparison to the controls.

Our data show that the type of liver damage may determine levels of DMEs in the organ (Figure 4 and Figure 5, Tables S2 and S3). CYP2C9 and CYP2C19 remained stable in all liver pathological states, and in comparable levels to the controls. As for the UGTs, protein levels of UGT1A1, UGT1A3 and UGT2B15 were also not changed. All other investigated enzymes demonstrated a downregulation trend. CYP1A1 abundance was markedly reduced in cholestatic pathologies, i.e., PBC (to 0.03% of the controls) and PSC (to 0.04% of the controls). In PBC, apart from CYP1A1, a marked downregulation was observed for CYP2B6 (to 44% of mean value of the controls), CYP2C8 (to 28% of the controls), CYP2E1 (to 39% of the controls) and CYP3A4 (to 47% of the controls). HCV pathology entailed prominent decrease in CYP2E1 (to 52% of the controls) and UGT2B7 (to 49% of the controls) abundances. ALD was characterized with significant reduction of protein abundance of CYP1A2 (to 33% of the controls), CYP2C8 (to 32% of the controls), CYP2D6 (to 14% of the controls), CYP2E1 (to 35% of the controls), CYP3A4 (to 27% of the controls) and UGT2B7 (to 47% of the controls). In AIH no significant changes in the studied enzymes protein levels were determined. 

**Figure 6:**
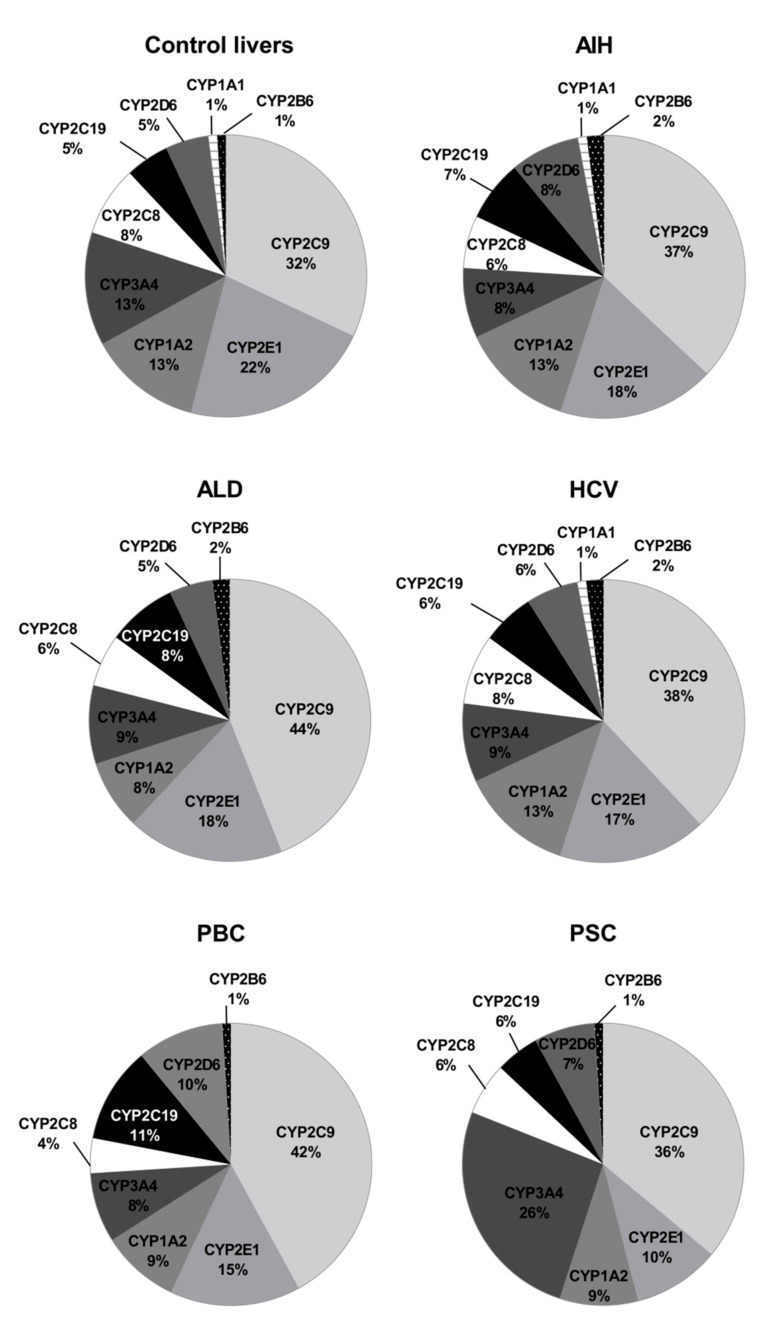
The pie chart of the individual enzyme proteins in livers tissues from hepatitis C (HCV), primary biliary cholangitis (PBC), primary sclerosing cholangitis (PSC), alcoholic liver disease (ALD) and autoimmune hepatitis (AIH) patients and the controls. The pie charts show the abundance of each enzymatic protein as a percentage of the sum of all enzyme proteins abundance.

The rank order of the studied CYPs was markedly affected by the studied liver pathologies. Figure 6 shows percentage contributions of all investigated CYP proteins stratified according to the different types of liver disease. In parallel to our analysis stratified by the Child–Pugh score, CYP2C9 showed highest ((32–44%) and CYP2B6 (1–2%) the lowest protein amounts in all studied liver pathologies.

In the control samples, for most of the studied DMEs, except for CYP2C19 and CYP2E1, significant, strong (rs > 0.6) correlations between mRNA expression and protein abundance (for CYP2D6 moderate) were observed. Liver pathologies affected the level of significant positive correlations. However, in AIH and to a lesser extent in PBC multiple positive correlations were still found (Table 1).

### 3.3. Genotyping

The genotyping studies of CYP2C19*, CYP2D6* and CYP3A5* resulted in the exclusion of the patient samples, who were genetically determined with the enzyme deficiency, i.e., for CYP2C19—2 controls, and for CYP2D6—1 control as well as AIH—1, HCV—1 and PBC—1 sample. In the case of CYP3A5, expresser status (*1/*3) was found in 12 subjects: 3 controls, 3 AIH, 1 ALD, 3 HCV, 1 PBC and 1 PSC. In all expressers, protein abundance exceeded 200 fmol/mg tissue, except for a PSC patient with the protein level of 46 fmol/mg tissue; in non-expressers CYP3A5 abundance ranged from 0 to 102.54 fmol/mg tissue (mean 26.21 fmol/mg tissue).

## 4. Discussion

It is known that impaired hepatic function may affect drug pharmacokinetics, and in turn efficacy and safety of drug therapy. However, due to ethical aspects and organizational issues, there is limited information about drug pharmacokinetics in liver diseases, especially in advanced stages. The published clinical pharmacokinetic studies suggest that liver failure can affect CYP1A2, CYP2C19, CYP2D6, CYP2E1 and CYP3A4 activities [1,2,3]. However, the estimation of precise contribution of the enzymes to overall altered drug kinetics in patients with deteriorated liver function is not possible due to a complex pathophysiology. Proteomic data on CYPs and UGTs offer a possibility to better characterize DMEs status in the liver, and to cover the gaps of quantitative information enabling construction of more adequate PBPK models of liver pathologies.

The present study provides for the first time precise quantitative data on the protein abundance of DMEs in the livers of patients with a wide spectrum of liver diseases stratified according to the Child–Pugh score. The study findings evidence that progression of liver dysfunction is associated with constant deterioration of most of the studied enzymes. However, protein abundances of CYP1A1, CYP2B6, CYP2C19 and CYP2D6 as well as UGT1A1, UGT1A3 and UGT2B15 did not differ significantly among the Child–Pugh class A, B and C livers. These observations are supported by the debrisoquine (CYP2D6 substrate) pharmacokinetic study, whose metabolism was found to be not affected by Child–Pugh class A and B liver disease [2]. However, the decrease in the apparent oral clearance of S-mephenytoin (CYP2C19 substrate) along with a corresponding reduction in the urinary excretion of the metabolite 4′-hydroxymephenytoin (suggesting its decreased metabolism) is contrary to the findings from the present study [1,2]. The discrepancy between the studies could be related to the reduced hepatocyte volume in the liver, and thus the diminished total liver CYP2C19 metabolic capacity developing with liver failure progression [18], as well as to a genetic status of the subjects (the patients of Adedoyin et al. [2], were phenotyped and poor metabolizers were excluded from the study, but as stated above apart from quantitative changes of the enzymatic protein abundance many other factors can affect pharmacokinetics in liver failure patients; our study excluded poor metabolizers of CYP2C19 and CYP2D6). The immunoblot-based, semiquantitative determination of CYP2C in livers, which is a less reliable assay than LC-MS/MS method due to its methodological limitations, classified as the A and B (according to the Child–Pugh) class showed rather stable protein levels (with the exception of cholestatic livers in one study) [13,14]. 

There are no literature references for CYP1A1 and CYP2B6 abundances, and the present study for the first time provides information about stable protein levels of these enzymes in liver pathology states, as the same protein abundances for the controls and for the livers of the Child–Pugh class from A to C were observed.

The present study revealed significant downregulation of the protein abundance of CYP1A2, which is in agreement with semiquantitative, antibody-based observations of George et al. and Guengerich et al. [13,14]. The protein quantitative data are supported by clinical findings, which revealed an altered caffeine (CYP1A2 substrate) pharmacokinetics in liver diseases. Frye et al. observed a 69% significantly lower caffeine metabolic ratio in decompensated liver disease (Pugh score ≥ 6), and no pharmacokinetic effects of the compensated disease (Pugh score = 5) [1]. 

The progression of liver disease also entailed a decrease in protein abundance of CYP2E1, which significant reduction was observed even in the Child–Pugh class A patients (as the unique downregulated enzyme levels in the early stage of liver failure were seen). These quantitative data are in agreement with the immunoblot CYP2E1 protein determination in cholestatic livers and cirrhotic livers [13,14]. The observed reduction of CYP2E1 abundance may contribute to 40% lower chlorzoxazone (CYP2E1 substrate) metabolic ratio observed in patients with moderate-severe liver disease [1]. 

Contradictory to the present study findings, non-significantly altered levels of CYP3A abundance (measured by an immunoblot technique, without discrimination of CYP3A4 and CYP3A5 isoforms) were provided by George et al. (for cholestatic liver diseases) and Guengerich et al. [13,14]. However, CYP3A enzymes were significantly affected by the non-cholestatic form of liver diseases [14]. Likewise in the case of CYP2C19, these studies did not consider highly polymorphic nature of CYP3A5, which might result in inconsistent observations. However, the present study findings are in keeping with clinical observations on midazolam pharmacokinetics, which demonstrated significantly 38% higher oral bioavailability and 41% lower total clearance of the drug (and unchanged plasma protein binding and distribution) in patients with chronic liver disease [3]. In our study, we did not analyze CYP3A5, as only few cases of expressers were found upon genotyping. However, we found that CYP3A5 expressers in different liver pathologies demonstrated protein abundance exceeding 200 fmol/mg liver tissue (except for a PSC patient with the protein level of 46 fmol/mg). A common CYP3A5*3 allele results in a cryptic splice site in intron 3, and an altered mRNA splicing and truncated non-functional protein in about 90% of Caucasian population, but the status of CYP3A5-expressor can affect drug pharmacokinetics (i.e., tacrolimus, midazolam, and possibly statins) in patients with the functional CYP3A5*1 allele [19].

There is no information about the disease-related changes of UGT2B7 protein abundance, but the downregulation observed in our study is also supported by significantly lower mRNA expression level of UGT2B7 in human liver diseases, which is also consistent with our mRNA expression findings for the enzyme [20]. 

The present study provides also for the first-time proteomic characteristics of CYP2C8 and CYP2C9 in different stages of liver diseases. The levels of both enzymes were reduced stepwise down along the liver diseases severity. 

The analysis of the liver samples according to the Child–Pugh class suggests that the most vulnerable enzyme was CYP2E1, which was downregulated in class A livers. The class B livers were characterized by a decrease in CYP2E1 and then CYP2C8, CYP3A4 as well as UGT2B7 abundances. In the Child–Pugh class C, the following enzymes were affected: CYP2E1, CYP2C8, CYP3A4 and CYP1A2, CYP2C9. These findings are only in part consistent with pharmacokinetic observations with substrates for CYP1A2, CYP2C9, CYP2D6 and CYP2E1. The reported pharmacokinetic study suggested that CYP2E1 was the most stable enzyme, whereas CYP2C19 (with CYP1A1 and CYP2D6 in the middle) was the most vulnerable in liver failure [1]. As stated above, lack of genetic information in the study of Frye et al. [1] could contribute to the reported discrepancies. The Child–Pugh scoring criteria are well-established and widely used to classify severity of liver dysfunction/cirrhosis, and the score parameters refer to hepatocyte function (albumin and bilirubin levels, prothrombin time), as well as to overall disease progression (encephalopathy and ascites; and these are more subjective) [21,22]. The Child–Pugh score is the most commonly used scale for assessing hepatic impairment in the case of drugs submitted for US FDA approval [23].

A pediatric population study on hepatic cytochrome P450 activity in livers, mainly with biliary atresia (explanted before transplantation), are mostly in keeping with the present study observations. A negative correlation of CYP1A2, CYP2C9, CYP2C19, CYP2D6, CYP2E1 and CYP3A4 activity with PELD score (pediatric end-stage liver disease score, range from 0 to 38) used to estimate relative disease severity and survival of patients awaiting liver transplantation was reported by de Bock et al. [24]. This study excluded poor metabolizers and gain-of-function subjects (CYP2C9, CYP2C19 and CYP2D6) after genotyping.

In the present study, an analysis of the specific type of liver disease and DMEs (both at mRNA and protein levels) was performed. Similar to other studies, some strong correlations between mRNA expression and protein levels could be found in normal livers [25]. However, liver pathological states affected the correlations, especially in PSC, ALD and HCV samples, which may suggest that the available mRNA data not reliably represent biological role of the enzymes. Only moderate correlations between cytochrome P450 mRNA expressions and corresponding cytochrome P450 activities were found for CYP2C19, CYP2D6 and CYP4A11 (rs = 0.44–0.56) in the intestine [26]. However, strong correlations (rs > 0.6) between hepatocyte protein levels and enzymatic activity were documented for CYP1A2, CYP2B6, CYP2D6 and CYP3A4 (of the studied relationships, a mild correlation was found for CYP2C9, rs = 0.42) [27]. Direct comparison of mRNA and protein levels with enzymatic activity reported by Ohtsuki et al. revealed that except for CYP2B6, a better correlation of enzyme activity with protein abundance (CYP1A2, CYP2C8, CYP2C9, CYP2C19, CYP2D6, CYP2E1, CYP4A11, CYP3A4) than with mRNA expression [25]. Thus, studies based only on transcriptome analysis do not always reflect protein enzyme abundances. Therefore, it seems that quantitative proteomic information may better reflect functional state of the enzymes. 

The study provides also information about DMEs abundance in different pathological states of the liver, i.e., viral—hepatitis C (HCV), toxic—alcoholic liver disease (ALD), immune/inflammatory—autoimmune hepatitis (AIH) and cholestatic—primary biliary cholangitis (PBC) and primary sclerosing cholangitis (PSC). Only one previous report has used a similar method to quantify hepatic drug metabolizing enzymes, but only in ALD and HCV-induced liver damage (without stratification to liver functional state) [12]. In ALD, the present study revealed stable protein levels of CYP1A1, CYP2B6, CYP2C9, CYP2C9, UGT1A1, UGT1A3 and UGT2B15 as well as downregulation of CYP1A2, CYP2C8, CYP2D6, CYP2E1, CYP3AA4 and UGT2B7. These results corroborate observations of Prasad et al., who documented reduction in protein abundance of CYP1A2, CYP2C8, CYP2E1, CYP3A4 and UGT2B7 in ALD livers [12]. However, contrary to our findings, downregulations of CYP2C9, CYP2D6, UGT2B15 protein levels were also observed. As for HCV pathology, the concordance was noted for CYP2D6 (in both studies not changed), CYP2E1 and UGT2B7 (in both studies downregulated), but reduction in CYP1A2, CYP2C9 and UGT2B15 was not reproduced. The observed discrepancies may be explained by different methodical procedures of sample preparation. While Prasad et al. isolated and analyzed the S9 fraction [12], the FASP procedure (eliminating any additional centrifugation steps that may cause loss of proteins) was used in the present study. In addition to other methodical differences (e.g., the use of different peptides), analysis of samples from different stages of liver disease might also contribute to the study results. 

The present study demonstrates a significant decline of CYP1A2 and CYP2C9 abundances in the course of progression of liver disease, and similar trend is observed for UGT2B15). Liver samples analyzed by Prasad et al. were characterized as end-stage liver disease, and the authors assumed that they represented the Child–Pugh score C, as livers explanted during liver transplantation were analyzed [12]. However, transplantation protocols qualify even the Child–Pugh score A subjects to the procedure, depending on the patient clinical picture. The published clinical pharmacokinetic studies involved patients, who were characterized only as “having liver disease” (without etiology), and those published are in keeping with the merged LC-MS/MS proteomic studies results for the following enzymatic proteins in ALD: CYP1A2 (caffeine), CYP2E1 (chlorzoxazone) and CYP3A4 (midazolam) as well as in HCV: CYP2E1 (chlorzoxazone) and CYP2D6 (debrisoquine) [1,2,3]. 

The present study provides also for the first-time quantitative information about DMEs in cholestatic liver disease, i.e., primary biliary cholangitis (intrahepatic cholestasis) and primary sclerosing cholangitis (extrahepatic cholestasis). In both cholestatic pathologies, marked downregulation of CYP1A1 abundance was noted, and in the case of PBC, significantly lower levels of CYP2B6, CYP2C8, CYP2E1 and CYP3A4 were also noted. Reduced CYP2E1 levels were also observed in cholestatic livers examined by George et al. (using a semiquantitative blot method) [14]. 

The study also reports new findings on DMEs profile in autoimmune hepatitis, where only downregulation trend of the studied enzymes was documented (except for UGT1A1 and UGT1A3), but without reaching statistical significance (*p* > 0.05). So, AIH seems to be the liver pathological state with a relatively stable function of DMEs.

Small number of PBC and PSC samples belongs to the study limitations, which should be considered when interpreting data. However, information about gene expression and protein content in these cholestatic diseases has not been published yet (the prevalence of both diseases is very low), and these data can be considered as preliminary. Patients medication could also affect the present study results. The liver diseases medication standards were modified during the period of the liver samples harvesting, and treatment approaches are also liver pathology and functional state dependent. Therefore, due to a limited number of samples for a given liver pathology and functional state, medication analysis was not reliable. However, patients with co-morbidities were excluded. A liver harvesting of the samples could also pose some bias. This study, likewise other reports [12,13,14], includes livers from patients with the organ failure (including cirrhosis), involving progressing changes in a number of hepatocytes (reduction), and other components of liver structure, i.e., sinusoidal endothelial cells, Kupffer cells, and hepatic stellate cells, fibroblasts and transdifferentiated myofibroblasts and fibrous connective tissue (expansion) [28]. The functional liver mass in the Child–Pugh class A, B, and C was shown to be 69%, 55%, and 28%, respectively, as compared with the control samples [29]. To limit the tissue harvesting bias, efforts were made to dissect only the parenchymal/functional, hepatocyte rich liver tissue. The interpersonal variability in the studied enzyme protein abundance observed in the present study (from CYP2C9 5.5-fold to CYP1A1 33-fold in controls), which is consistent with data of Othsuki et al. [25], Achour et al. [27], Kawakami et al. [30], could also affect the data analysis outcome (to reduce impact of genetic factors poor metabolizers of CYP2C19 and CYP2D6 as well as CYP3A5 expressers were excluded from the analysis). 

As already described in the Introduction, there are few studies available investigating impact of liver diseases on protein abundances of clinically relevant DMEs. The novelty of the study is represented by a variety of liver diseases evaluated, and in parallel the analysis of the samples according to the frequently used Child–Pugh score. The quantitative information about DMEs protein abundance can be implemented to PBPK models to calculate the clinical relevance in terms of the liver metabolic capacity. However, the application of the method itself (protein quantification) in diagnostics is not expected to be a clinical routine because the procedure is expensive, time-consuming and requires a certain level of methodical expertise. 

## 5. Conclusions

In conclusion, it can be stated that the study provides the information about DMEs proteomic quantitative status in five liver pathologies of different etiology, i.e., hepatitis C, alcoholic liver disease, primary biliary cholangitis, primary sclerosing cholangitis and autoimmune hepatitis. ALD and PSC involved the most prominent changes in protein abundances of DMEs, with 6 and 5 significantly downregulated proteins (out of 13 studied), respectively. It seems that CYP2E1 is the most vulnerable enzyme, as it was the only one with markedly reduced protein levels in the Child–Pugh class A livers. The protein abundance of CYP1A1, CYP2B6, CYP2C19, CYP2D6 as well as UGT1A1, UGT1A3 and UGT2B15 was relatively stable in the course of progression of liver function deterioration. The results from the present study may be applied to optimize the existing in vitro in vivo extrapolation of drug metabolism in PBPK models. Moreover, information on disease-related changes in the hepatic protein abundance of DMEs may allow more precise predictions on drug pharmacokinetics and drug–drug interactions in patients suffering from different forms of liver disease and in turn appropriate dose-adjustments. 

## Figures and Tables

**Table 1:** Correlation (Spearman coefficient, r) between protein and mRNA levels of the P450s and UGTs in human livers stratified by the liver pathology.

mRNA vs. Protein Correlation Coefficient						
Protein	Control *n* = 20 ^a^	AIH *n* = 20 ^b^	ALD *n* = 20	HCV *n* = 21 ^c^	PBC *n* = 10 ^d^	PSC *n* = 6
**CYP1A1**	0.664 ***	0.722 ***	0.443	0.736 ***	0.225	−0.393
**CYP1A2**	0.824 ***	0.857 ***	0.595 **	0.766 ***	0.867 **	1.000 **
**CYP2B6**	0.612 **	0.573 **	−0.036	0.394	0.900 ***	−0.257
**CYP2C8**	0.645 **	0.588 **	0.140	0.182	0.842 **	−0.086
**CYP2C9**	0.620 **	0.686 ***	0.498 *	0.347	0.867 **	0.829 *
**CYP2C19**	0.325	−0.038	−0.368	0.099	0.224	0.029
**CYP2D6**	0.586 **	0.614 **	0.105	0.470 *	0.600	0.086
**CYP2E1**	0.352	0.580 **	0.499 *	0.355	0.479	0.371
**CYP3A4**	0.889 ***	0.722 ***	0.714 ***	0.712 ***	0.890 ***	0.543
**UGT1A1**	0.675 **	0.684 ***	0.383	0.504 *	0.927 ***	0.143
**UGT1A3**	0.699 ***	0.453 *	−0.341	0.451 *	0.612	0.771
**UGT2B15**	0.800 ***	0.453 *	0.229	0.479 *	0.467	0.371
**UGT2B7**	0.725 ***	0.740 ***	0.505 *	0.264	0.782 **	0.829 *

* *p* < 0.05; ** *p* < 0.01; *** *p* < 0.001; ^a^ CYP2C19 *n* = 18, CYP2D6 *n* = 19; ^b^ CYP2D6 *n* = 19; ^c^ CYP2D6 *n* = 19; ^d^ CYP2D6 *n* = 9.

## Data Availability

Not applicable.

## Supplementary Materials

The following are available online at https://www.mdpi.com/article/10.3390/pharmaceutics13091334/s1, Table S1: Characteristics of the subjects. Table S2: The mRNA (messenger RNA) expression (relative to the mean value of the control group) of the CYPs and UGTs in different liver pathologies (AIH, ALD, HCV, PBC, PSC) and disease stages (Child–Pugh class A, B and C) as well as in the controls. Table S3: Protein abundance (fmol/mg tissue) of the CYPs and UGTs in different liver pathologies (AIH, ALD, HCV, PBC, PSC) and disease stages (Child–Pugh class A, B and C) as well as in the controls. Table S4: Differences in the drug metabolizing enzymes protein abundance and mRNA levels between studied groups.
