# Supplementary material for: Gene Expression and Protein Abundance of Hepatic Drug Metabolizing Enzymes in Liver Pathology
Source: Pharmaceutics. 2021 Aug 25;13(9):1334. doi: 10.3390/pharmaceutics13091334 (PMC8471929; doi:10.3390/pharmaceutics13091334)
Supplement: Supplementary file 1 [file pharmaceutics-13-01334-s001.zip › pharmaceutics-1317942-supplementary.pdf]

pharmaceutics

MDPI

Supplementary Materials: Gene Expression and Protein Abundance of Hepatic Drug Metabolizing Enzymes in Liver Pathology

Marek Drozdzik, Joanna Lapczuk-Romanska, Christoph Wenzel, Sylwia Szelag-Pieniek, Mariola Post, Łukasz Skalski, Mateusz Kurzawski and Stefan Oswald

Supplementary information

Table S1. Characteristics of the subjects (mean±SD).

Parameter/disease	Controls *n*=20	HCV *n*=21	PBC *n*=10	PSC *n*=6	ALC *n*=20	AIH *n*=20
Sex [male/female]	11/9	10/11	1/9	4/2	16/4	8/12
Age [years]	63 ± 10	52 ± 5	59 ± 4	43 ± 10	51 ± 6	47 ± 16
Child-Pugh [A/B/C]	-	7/10/4	2/4/4	3/3/0	0/8/12	6/6/8
Total bilirubin [mg/dl]	0.59 ± 0.25	2.38 ± 1.37	6.42 ± 6.72	8.14 ± 8.14	4.4 ± 4.02	3.54 ± 3.53
Albumin [g/dl]	3.89 ± 0.38	3.31 ± 0.45	3.13 ± 0.65	3.7 ± 0.44	3.03 ± 0.50	3.29 ± 0.39
PT [s]	12.7 ± 2.3	14.4 ± 2.0	12.5 ± 1.2	13.2 ± 2.8	16.0 ± 2.2	14.6 ± 2.5
INR	1.14 ± 0.21	1.39 ± 0.27	1.19 ± 0.21	1.4 ± 0.52	1.47 ± 0.23	1.42 ± 0.41

HCV—hepatitis C, PBC—primary biliary cholangitis, PSC—primary sclerosing hepatitis, ALC—alcoholic cirrhosis and AIH—autoimmune hepatitis, PT—prothrombin time, INR—International Normalized Ratio

Table S2. The mRNA expression (relative to the mean value of the control group- $2^{-\Delta\Delta Ct}$ method) of the P450s and UGTs in different liver pathologies (HCV—hepatitis C. PBC—primary biliary cholangitis. PSC—primary sclerosing cholangitis. ALD—alcoholic liver disease. AIH—autoimmune hepatitis) and disease stages (Child–Pugh class A. B and C) as well as in the controls.

	Child-Pugh class				
	**A**	**B**	**C**	**A+B+C**	**Controls**
			**CYP1A1**		
AIH	*n*=6<br>0.95 (0.09–5.45)<br>1.74 ± 1.97 (113%)	*n*=6<br>0.45 (0.18–1.05)<br>0.52 ± 0.35 (66%)	*n*=8<br>0.52 (0.00–4.42)<br>1.04 ± 1.52 (145%)	*n*=20<br>0.59 (0.00–5.45)<br>1.10 ± 1.46 (133%)	
ALD	–	*n*=8<br>0.24 (0.06–1.41)<br>0.44 ± 0.48 (109%)	*n*=12<br>0.32 (0.07–1.87)<br>0.65 ± 0.63 (98%)	*n*=20<br>0.31 (0.06–1.87)<br>0.56 ± 0.57 (101%)	
HCV	*n*=7<br>0.38 (0.16–0.68)<br>0.39 ± 0.18 (45%)	*n*=10<br>1.44 (0.32–4.43)<br>1.86 ± 1.52 (82%)	*n*=4<br>0.57 (0.11–1.70)<br>0.74 ± 0.72 (98%)	*n*=21<br>0.59 (0.11–4.43)<br>1.15 ± 1.27 (110%)	
PBC	*n*=2<br>0.33 (0.17–0.49)<br>0.33 ± 0.23 (70%)	*n*=4<br>0.66 (0.52–0.83)<br>0.67 ± 0.16 (24%)	*n*=4<br>0.17 (0.06–0.76)<br>0.29 ± 0.32 (108%)	*n*=10<br>0.51 (0.06–0.83)<br>0.45 ± 0.29 (64%)	
PSC	*n*=3	*n*=3	-	*n*=6	

	0.44 (0.25–0.48)<br>0.39 ± 0.12 (32%)	0.28 (0.24–0.31)<br>0.27 ± 0.04 (13%)		0.29 (0.24–0.48)<br>0.33 ± 0.10 (31%)	
All diseases	*n*=18<br>0.47 (0.09–5.45)<br>0.83 ± 1.26 (152%)	*n*=31<br>0.52 (0.06–4.43)<br>0.93 ± 1.10 (119%)	*n*=28<br>0.32 (0.00–4.42)<br>0.72 ± 0.94 (130%)	*n*=77<br>0.48 (0.00–5.45)<br>0.83 ± 1.07 (129%)	
Controls					*n*=20<br>0.39 (0.15–4.94)<br>1.00 ± 1.25 (125%)
			**CYP1A2**		
AIH	*n*=6<br>0.47 (0.01–3.35)<br>0.98 ± 1.24 (127%)	*n*=6<br>0.45 (0.09–1.12)<br>0.52 ± 0.44 (84%)	*n*=8<br>0.27 (0.00–2.40)<br>0.49 ± 0.80 (162%)	*n*=20<br>0.45 (0.00–3.35)<br>0.65 ± 0.86 (133%)	
ALD	–	*n*=8<br>0.22 (0.07–0.94)<br>0.35 ± 0.33 (95%)	*n*=12<br>0.21 (0.01–1.33)<br>0.32 ± 0.37 (114%)	*n*=20<br>0.21 (0.01–1.33)<br>0.33 ± 0.34 (104%)	
HCV	*n*=7<br>0.21 (0.17–1.26)<br>0.46 ± 0.43 (93%)	*n*=10<br>0.74 (0.23–1.68)<br>0.75 ± 0.46 (61%)	*n*=4<br>0.24 (0.00–1.28)<br>0.44 ± 0.60 (136%)	*n*=21<br>0.42 (0.00–1.68)<br>0.60 ± 0.48 (80%)	
PBC	*n*=2<br>0.52 (0.38–0.67)<br>0.52 ± 0.20 (39%)	*n*=4<br>0.33 (0.12–0.56)<br>0.33 ± 0.20 (59%)	*n*=4<br>0.06 (0.01–0.75)<br>0.22 ± 0.36 (162%)	*n*=10<br>0.31 (0.01–0.75)<br>0.33 ± 0.27 (83%)	
PSC	*n*=3<br>0.56 (0.26–0.91)<br>0.58 ± 0.32 (56%)	*n*=3<br>0.35 (0.18–0.90)<br>0.48 ± 0.37 (78%)	–	*n*=6<br>0.45 (0.18–0.91)<br>0.53 ± 0.32 (60%)	
All diseases	*n*=18<br>0.42 (0.01–3.35)<br>0.66 ± 0.77 (116%)	*n*=31<br>0.36 (0.07–1.68)<br>0.52 ± 0.41 (78%)	*n*=28<br>0.20 (0.00–2.40)<br>0.37 ± 0.53 (143%)	*n*=77<br>0.33 (0.00–3.35)<br>0.50 ± 0.56 (111%)	
Controls					*n*=20<br>0.85 (0.18–3.00)<br>1.00 ± 0.68 (68%)
			**CYP2B6**		
AIH	*n*=6<br>0.24 (0.10–0.92)<br>0.37 ± 0.31 (43%)	*n*=6<br>0.19 (0.10–1.44)<br>0.41 ± 0.52 (126%)	*n*=8<br>0.21 (0.00–1.96)<br>0.39 ± 0.64 (163%)	*n*=20<br>0.21 (0.00–1.96)<br>0.39 ± 0.50 (127%)	
ALD	–	*n*=8<br>0.70 (0.24–2.27)<br>0.87 ± 0.69 (79%)	*n*=12<br>0.59 (0.13–1.87)<br>0.77 ± 0.53 (69%)	*n*=20<br>0.66 (0.13–2.27)<br>0.81 ± 0.58 (72%)	
HCV	*n*=7<br>1.25 (0.56–4.24)<br>1.47 ± 1.28 (87%)	*n*=10<br>1.05 (0.90–2.06)<br>1.21 ± 0.39 (32%)	*n*=4<br>0.83 (0.27–4.36)<br>1.57 ± 1.90 (121%)	*n*=21<br>1.06 (0.27–4.36)<br>1.37 ± 1.06 (78%)	
PBC	*n*=2<br>0.27 (0.23–0.30)<br>0.27 ± 0.05 (18%)	*n*=4<br>0.43 (0.11–0.63)<br>0.40 ± 0.22 (54%)	*n*=4<br>0.32 (0.07–0.99)<br>0.42 ± 0.44 (105%)	*n*=10<br>0.35 (0.07–0.99)<br>0.38 ± 0.29 (76%)	
PSC	*n*=3<br>1.07 (0.73–1.55)<br>1.12 ± 0.41 (37%)	*n*=3<br>0.54 (0.19–0.65)<br>0.46 ± 0.24 (53%)	–	*n*=6<br>0.69 (0.19–1.55)<br>0.79 ± 0.47 (60%)	
All diseases	*n*=18<br>0.71 (0.10–4.24)<br>0.91 ± 0.95 (105%)	*n*=31<br>0.68 (0.10–2.27)<br>0.79 ± 0.57 (73%)	*n*=28<br>0.44 (0.00–4.36)<br>0.73 ± 0.89 (123%)	*n*=77<br>0.56 (0.00–4.36)<br>0.80 ± 0.79 (99%)	
Controls					*n*=20

					0.95 (0.07–2.90)<br>1.00 ± 0.65 (65%)
**CYP2C8**					
AIH	*n*=6<br>0.68 (0.33–1.12)<br>0.71 ± 0.28 (39%)	*n*=6<br>0.55 (0.21–0.96)<br>0.58 ± 0.26 (45%)	*n*=8<br>0.59 (0.00–1.64)<br>0.64 ± 0.55 (86%)	*n*=20<br>0.63 (0.00–1.64)<br>0.65 ± 0.39 (60%)	
ALD	–	*n*=8<br>0.39 (0.13–1.92)<br>0.66 ± 0.63 (96%)	*n*=12<br>0.48 (0.28–1.02)<br>0.52 ± 0.22 (43%)	*n*=20<br>0.42 (0.13–1.92)<br>0.57 ± 0.43 (74%)	
HCV	*n*=7<br>0.87 (0.56–1.26) 0.88<br>± 0.22 (25%)	*n*=10<br>0.77 (0.41–1.40)<br>0.87 ± 0.34 (39%)	*n*=4<br>0.75 (0.08–1.24) 0.71<br>± 0.48 (67%)	*n*=21<br>0.79 (0.08–1.40) 0.84<br>± 0.33 (39%)	
PBC	*n*=2<br>0.55 (0.54–0.55) 0.55<br>± 0.00 (1%)	*n*=4<br>0.46 (0.21–0.70) 0.46<br>± 0.22 (47%)	*n*=4<br>0.23 (0.11–0.80) 0.34<br>± 0.31 (92%)	*n*=10<br>0.46 (0.11–0.80) 0.43<br>± 0.23 (55%)	
PSC	*n*=3<br>0.65 (0.57–1.30) 0.84<br>± 0.40 (48%)	*n*=3<br>0.91 (0.41–1.13) 0.81<br>± 0.37 (46%)	–	*n*=6<br>0.78 (0.41–1.30)<br>0.83 ± 0.35 (42%)	
All diseases	*n*=18<br>0.74 (0.33–1.30) 0.78<br>± 0.27 (34%)	*n*=31<br>0.59 (0.13–1.92) 0.70<br>± 0.42 (60%)	*n*=28<br>0.48 (0.00–1.64) 0.56<br>± 0.39 (69%)	*n*=77<br>0.60 (0.00–1.92) 0.67<br>± 0.38 (57%)	
Controls					*n*=20<br>1.01 (0.28–2.24) 1.00<br>± 0.45 (45%)
**CYP2C9**					
AIH	*n*=6<br>1.64 (0.39–2.93) 1.69<br>± 1.06 (63%)	*n*=6<br>1.48 (0.70–3.09) 1.61<br>± 0.90 (56%)	*n*=8<br>0.96 (0.00–5.67) 1.55<br>± 1.83 (117%)	*n*=20<br>1.14 (0.00–5.67) 1.61<br>± 1.32 (82%)	
ALD	–	*n*=8<br>0.65 (0.26–1.73) 0.85<br>± 0.54 (64%)	*n*=12<br>0.75 (0.24–1.16)<br>0.70 ± 0.28 (40%)	*n*=20<br>0.72 (0.24–1.73) 0.76<br>± 0.40 (52%)	
HCV	*n*=7<br>1.21 (1.00–1.57)<br>1.26 ± 0.23 (18%)	*n*=10<br>1.18 (0.66–1.96)<br>1.26 ± 0.39 (31%)	*n*=4<br>0.88 (0.12–1.53) 0.85<br>± 0.78 (92%)	*n*=21<br>1.18 (0.12–1.96)<br>1.18 ± 0.45 (38%)	
PBC	*n*=2<br>1.06 (0.91–1.21) 1.06<br>± 0.21 (20%)	*n*=4<br>0.64 (0.53–1.36) 0.79<br>± 0.39 (49%)	*n*=4<br>0.31 (0.11–0.99)<br>0.43 ± 0.40 (93%)	*n*=10<br>0.64 (0.11–1.36)<br>0.70 ± 0.42 (60%)	
PSC	*n*=3<br>1.14 (0.96–1.67) 1.26<br>± 0.37 (29%)	*n*=3<br>1.08 (0.65–1.20) 0.98<br>± 0.29 (30%)	–	*n*=6<br>1.11 (0.65–1.67) 1.12<br>± 0.33 (30%)	
All diseases	*n*=18<br>1.17 (0.39–2.93) 1.38<br>± 0.65 (47%)	*n*=31<br>1.08 (0.26–3.09) 1.13<br>± 0.60 (53%)	*n*=28<br>0.74 (0.00–5.67)<br>0.93 ± 1.08 (116%)	*n*=77<br>0.99 (0.00–5.67) 1.12<br>± 0.82 (74%)	
Controls					*n*=20<br>1.02 (0.27–1.49) 1.00<br>± 0.31 (31%)
**CYP2C19**					
AIH	*n*=6<br>0.61 (0.28–2.83) 0.92<br>± 0.95 (104%)	*n*=6<br>0.24 (0.03–0.45) 0.22<br>± 0.16 (71%)	*n*=8<br>0.17 (0.01–0.74) 0.22<br>± 0.25 (112%)	*n*=20<br>0.27 (0.01–2.83) 0.43<br>± 0.61 (143%)	

ALD	–	*n*=8<br>0.27 (0.15–1.30) 0.41 ± 0.38 (93%)	*n*=12<br>0.22 (0.10–0.68) 0.25 ± 0.15 (61%)	*n*=20<br>0.24 (0.10–1.30) 0.31 ± 0.27 (87%)	
HCV	*n*=7<br>0.32 (0.21–0.38) 0.30 ± 0.07 (22%)	*n*=10<br>0.35 (0.17–0.70) 0.38 ± 0.16 (43%)	*n*=4<br>0.56 (0.23–1.26) 0.65 ± 0.49 (76%)	*n*=21<br>0.32 (0.17–1.26) 0.40 ± 0.26 (64%)	
PBC	*n*=2<br>1.03 (0.68–1.39) 1.03 ± 0.50 (48%)	*n*=4<br>0.34 (0.20–1.59) 0.62 ± 0.66 (106%)	*n*=4<br>0.20 (0.15–0.24) 0.20 ± 0.04 (18%)	*n*=10<br>0.24 (0.15–1.59) 0.53 ± 0.53 (99%)	
PSC	*n*=3<br>0.80 (0.72–1.62) 1.05 ± 0.50 (48%)	*n*=3<br>0.58 (0.23–2.88) 1.23 ± 1.44 (117%)	–	*n*=6<br>0.76 (0.23–2.88) 1.14 ± 0.97 (85%)	
All diseases	*n*=18<br>0.46 (0.18–2.83) 0.66 ± 0.64 (97%)	*n*=31<br>0.28 (0.03–2.53) 0.43 ± 0.49 (114%)	*n*=28<br>0.21 (0.01–1.10) 0.28 ± 0.25 (90%)	*n*=77<br>0.26 (0.01–2.83) 0.43 ± 0.48 (112%)	
Controls					*n*=18<br>0.76 (0.15–2.36) 1.00 ± 0.78 (78%)
			**CYP2D6**		
AIH	*n*=6<br>1.43 (0.58–2.64) 1.51 ± 0.85 (56%)	*n*=5<br>0.29 (0.05–1.93) 0.70 ± 0.79 (113%)	*n*=8<br>0.36 (0.00–2.96) 0.86 ± 1.12 (130%)	*n*=20<br>0.56 (0.00–2.96) 0.99 ± 0.96 (96%)	
ALD	–	*n*=8<br>0.66 (0.20–1.42) 0.67 ± 0.41 (62%)	*n*=12<br>0.56 (0.24–1.01) 0.63 ± 0.23 (36%)	*n*=20<br>0.60 (0.20–1.42) 0.64 ± 0.31 (48%)	
HCV	*n*=7<br>0.93 (0.46–1.53) 1.01 ± 0.36 (35%)	*n*=8<br>0.95 (0.21–3.03) 1.10 ± 0.93 (85%)	*n*=4<br>0.51 (0.29–0.82) 0.53 ± 0.23 (42%)	*n*=19<br>0.87 (0.21–3.03) 0.95 ± 0.66 (70%)	
PBC	*n*=2<br>0.64 (0.55–0.72) 0.64 ± 0.12 (19%)	*n*=4<br>0.66 (0.33–2.28) 0.98 ± 0.88 (90%)	*n*=3<br>2.91 (0.27–2.97) 2.05 ± 1.54 (75%)	*n*=9<br>0.72 (0.27–2.97) 1.26 ± 1.12 (89%)	
PSC	*n*=3<br>1.21 (0.97–2.11) 1.43 ± 0.60 (42%)	*n*=3<br>0.79 (0.33–0.90) 0.67 ± 0.30 (45%)	–	*n*=6<br>0.93 (0.33–2.11) 1.05 ± 0.59 (57%)	
All diseases	*n*=18<br>0.96 (0.47–2.64) 1.22 ± 0.63 (52%)	*n*=28<br>0.67 (0.05–3.08) 0.85 ± 0.71 (84%)	*n*=27<br>0.54 (0.00–3.03) 0.85 ± 0.88 (104%)	*n*=73<br>0.73 (0.00–3.08) 0.94 ± 0.77 (82%)	
Controls					*n*=19<br>0.90 (0.29–1.98) 1.00 ± 0.49 (49%)
			**CYP2E1**		
AIH	*n*=6<br>0.54 (0.07–1.26) 0.62 ± 0.47 (76%)	*n*=6<br>0.47 (0.25–1.06) 0.52 ± 0.28 (55%)	*n*=8<br>0.37 (0.00–2.82) 0.66 ± 0.93 (141%)	*n*=20<br>0.48 (0.00–2.82) 0.60 ± 0.63 (105%)	
ALD	–	*n*=8<br>0.37 (0.07–0.85) 0.37 ± 0.24 (65%)	*n*=12<br>0.26 (0.06–1.00) 0.32 ± 0.28 (89%)	*n*=20<br>0.28 (0.06–1.00) 0.34 ± 0.26 (77%)	
HCV	*n*=7<br>0.43 (0.19–0.91) 0.54 ± 0.27 (50%)	*n*=10<br>0.58 (0.37–0.85) 0.64 ± 0.18 (27%)	*n*=4<br>0.32 (0.17–0.66) 0.37 ± 0.23 (63%)	*n*=21<br>0.54 (0.17–0.91) 0.55 ± 0.23 (42%)	

PBC	n=2<br>0.62 (0.42–0.81) 0.62 ± 0.27 (45%)	n=4<br>0.48 (0.39–0.49)<br>0.46 ± 0.05 (10%)	n=4<br>0.20 (0.12–0.31)<br>0.21 ± 0.08 (39%)	n=10<br>0.41 (0.12–0.81)<br>0.39 ± 0.20 (51%)	
PSC	n=3<br>0.33 (0.25–0.38) 0.32 ± 0.07 (21%)	n=3<br>0.33 (0.15–0.64) 0.37 ± 0.25 (67%)	–	n=6<br>0.33 (0.15–0.64) 0.35 ± 0.17 (48%)	
All diseases	n=18<br>0.42 (0.07–1.26) 0.54 ± 0.33 (61%)	n=31<br>0.47 (0.07–1.06)<br>0.50 ± 0.23 (47%)	n=28<br>0.25 (0.00–2.82) 0.41 ± 0.54 (133%)	n=77<br>0.39 (0.00–2.82) 0.47 ± 0.39 (82%)	
Controls					n=20<br>1.01 (0.33–1.56) 1.00 ± 0.37 (37%)
			**CYP3A4**		
AIH	n=6<br>0.64 (0.42–1.06) 0.67 ± 0.21 (31%)	n=6<br>0.82 (0.40–1.95) 0.93 ± 0.56 (60%)	n=8<br>0.50 (0.00–11.02)<br>1.83 ± 3.76 (206%)	n=20<br>0.62 (0.00–11.02)<br>1.21 ± 2.37 (195%)	
ALD	–	n=8<br>0.31 (0.12–1.02) 0.46 ± 0.35 (76%)	n=12<br>0.28 (0.07–1.04) 0.37 ± 0.28 (77%)	n=20<br>0.29 (0.07–1.04) 0.40 ± 0.31 (76%)	
HCV	n=7<br>0.60 (0.17–1.50) 0.75 ± 0.48 (64%)	n=10<br>0.71 (0.25–1.74)<br>0.72 ± 0.44 (62%)	n=4<br>0.74 (0.00–2.56) 1.01 ± 1.22 (121%)	n=21<br>0.62 (0.00–2.56) 0.78 ± 0.63 (80%)	
PBC	n=2<br>1.37 (1.03–1.70)<br>1.37 ± 0.47 (35%)	n=4<br>0.35 (0.17–1.58) 0.61 ± 0.67 (109%)	n=4<br>0.04 (0.01–0.26)<br>0.09 ± 0.12 (132%)	n=10<br>0.22 (0.01–1.70) 0.55 ± 0.65 (117%)	
PSC	n=3<br>1.85 (0.51–2.20) 1.52 ± 0.89 (59%)	n=3<br>0.94 (0.71–5.56)<br>2.40 ± 2.73 (114%)	–	n=6<br>1.40 (0.51–5.56)<br>1.96 ± 1.88 (96%)	
All diseases	n=18<br>0.66 (0.17–2.20) 0.92 ± 0.57 (62%)	n=31<br>0.62 (0.12–5.56) 0.84 ± 0.99 (118%)	n=28<br>0.25 (0.00–11.02)<br>0.84 ± 2.09 (249%)	n=77<br>0.53 (0.00–11.02)<br>0.86 ± 1.42 (165%)	
Controls					n=20<br>0.87 (0.07–2.45)<br>1.00 ± 0.70 (70%)
			**UGT1A1**		
AIH	n=6<br>0.60 (0.09–4.50) 1.42 ± 1.72 (121%)	n=6<br>0.31 (0.18–2.05)<br>0.72 ± 0.77 (107%)	n=8<br>0.88 (0.00–1.63) 0.83 ± 0.67 (81%)	n=20<br>0.48 (0.00–4.50) 0.97 ± 1.09 (112%)	
ALD	–	n=8<br>1.06 (0.13–2.18) 1.05 ± 0.74 (71%)	n=12<br>1.13 (0.45–1.99) 1.15 ± 0.53 (46%)	n=20<br>1.07 (0.13–2.18) 1.11 ± 0.60 (54%)	
HCV	n=7<br>1.22 (0.27–1.90) 1.16 ± 0.53 (46%)	n=10<br>1.11 (0.37–3.04) 1.30 ± 0.71 (55%)	n=4<br>0.60 (0.30–4.40) 1.48 ± 1.96 (133%)	n=21<br>1.05 (0.27–4.40) 1.29 ± 0.95 (74%)	
PBC	n=2<br>0.73 (0.56–0.90)<br>0.73 ± 0.24 (33%)	n=4<br>0.96 (0.75–1.21)<br>0.97 ± 0.25 (25%)	n=4<br>0.37 (0.26–1.24) 0.56 ± 0.45 (82%)	n=10<br>0.76 (0.26–1.24) 0.76 ± 0.37 (48%)	
PSC	n=3<br>0.91 (0.45–1.01)<br>0.79 ± 0.30 (38%)	n=3<br>1.14 (1.07–1.26) 1.16 ± 0.10 (8%)	–	n=6<br>1.04 (0.45–1.26) 0.97 ± 0.29 (29%)	

All diseases	*n*=18<br>0.91 (0.09–4.50) 1.14 ± 1.03 (90%)	*n*=31<br>1.07 (0.13–3.04) 1.07 ± 0.66 (62%)	*n*=28<br>0.81 (0.00–4.40) 1.02 ± 0.87 (85%)	*n*=77<br>1.00 (0.00–4.50) 1.07 ± 0.82 (77%)	
Controls					*n*=20<br>0.86 (0.27–2.67) 1.00 ± 0.59 (59%)
			**UGT1A3**		
AIH	*n*=6<br>0.40 (0.17–0.94) 0.44 ± 0.29 (65%)	*n*=6<br>0.31 (0.15–0.89) 0.42 ± 0.29 (68%)	*n*=8<br>0.42 (0.00–6.75) 1.17 ± 2.27 (194%)	*n*=20<br>0.35 (0.00–6.75) 0.73 ± 1.44 (198%)	
ALD	–	*n*=8<br>0.44 (0.20–1.24) 0.52 ± 0.33 (63%)	*n*=12<br>0.36 (0.21–0.81) 0.44 ± 0.21 (48%)	*n*=20<br>0.39 (0.20–1.24) 0.47 ± 0.26 (55%)	
HCV	*n*=7<br>0.42 (0.25–1.05) 0.56 ± 0.28 (51%)	*n*=10<br>0.61 (0.35–1.05) 0.62 ± 0.22 (35%)	*n*=4<br>0.40 (0.12–0.89) 0.46 ± 0.36 (78%)	*n*=21<br>0.58 (0.12–1.05) 0.57 ± 0.26 (46%)	
PBC	*n*=2<br>0.66 (0.45–0.87) 0.66 ± 0.30 (45%)	*n*=4<br>0.54 (0.17–1.13) 0.59 ± 0.41 (68%)	*n*=4<br>0.48 (0.44–0.54) 0.49 ± 0.04 (9%)	*n*=10<br>0.48 (0.17–1.13) 0.56 ± 0.27 (47%)	
PSC	*n*=3<br>0.75 (0.25–1.26) 0.75 ± 0.51 (67%)	*n*=3<br>0.50 (0.49–1.23) 0.74 ± 0.43 (58%)	–	*n*=6<br>0.62 (0.25–1.26) 0.75 ± 0.42 (56%)	
All diseases	*n*=18<br>0.45 (0.17–1.26) 0.56 ± 0.32 (56%)	*n*=31<br>0.49 (0.15–1.24) 0.56 ± 0.30 (53%)	*n*=28<br>0.44 (0.00–6.75) 0.66 ± 1.22 (185%)	*n*=77<br>0.45 (0.00–6.75) 0.60 ± 0.77 (128%)	
Controls					*n*=20<br>0.81 (0.30–2.47) 1.00 ± 0.60 (60%)
			**UGT2B7**		
AIH	*n*=6<br>0.70 (0.35–2.41) 0.92 ± 0.77 (84%)	*n*=6<br>0.55 (0.00–1.12) 0.57 ± 0.46 (81%)	*n*=8<br>0.16 (0.00–1.96) 0.46 ± 0.67 (148%)	*n*=20<br>0.46 (0.00–2.41) 0.63 ± 0.65 (103%)	
ALD	–	*n*=8<br>0.39 (0.19–1.28) 0.57 ± 0.42 (74%)	*n*=12<br>0.48 (0.13–0.77) 0.45 ± 0.20 (45%)	*n*=20<br>0.43 (0.13–1.28) 0.50 ± 0.30 (61%)	
HCV	*n*=7<br>0.76 (0.48–1.13) 0.79 ± 0.22 (28%)	*n*=10<br>0.73 (0.26–1.02) 0.70 ± 0.20 (29%)	*n*=4<br>0.22 (0.03–1.30) 0.44 ± 0.59 (132%)	*n*=21<br>0.73 (0.03–1.30) 0.68 ± 0.32 (46%)	
PBC	*n*=2<br>0.58 (0.51–0.65) 0.58 ± 0.10 (18%)	*n*=4<br>0.50 (0.21–0.80) 0.50 ± 0.25 (49%)	*n*=4<br>0.20 (0.02–0.67) 0.27 ± 0.31 (115%)	*n*=10<br>0.47 (0.02–0.80) 0.43 ± 0.27 (63%)	
PSC	*n*=3<br>0.73 (0.64–1.45) 0.94 ± 0.44 (47%)	*n*=3<br>0.51 (0.44–0.82) 0.59 ± 0.20 (35%)	–	*n*=6<br>0.68 (0.44–1.45) 0.76 ± 0.36 (48%)	
All diseases	*n*=18<br>0.71 (0.35–2.41) 0.83 ± 0.48 (57%)	*n*=31<br>0.58 (0.00–1.28) 0.61 ± 0.32 (53%)	*n*=28<br>0.32 (0.00–1.96) 0.43 ± 0.43 (102%)	*n*=77<br>0.54 (0.00–2.41) 0.59 ± 0.43 (72%)	
Controls					*n*=20<br>0.92 (0.40–1.94) 1.00 ± 0.41 (41%)

			**UGT2B15**		
AIH	*n*=6<br>3.80 (0.62–5.16) 3.47<br>± 1.54 (44%)	*n*=6<br>0.90 (0.44–4.09) 1.66<br>± 1.56 (94%)	*n*=8<br>1.09 (0.02–11.34)<br>2.57 ± 3.76 (146%)	*n*=20<br>1.78 (0.02–11.34)<br>2.57 ± 2.65 (103%)	
ALD	–	*n*=8<br>0.69 (0.38–1.60) 0.82<br>± 0.43 (52%)	*n*=12<br>0.95 (0.68–1.91) 1.11<br>± 0.44 (40%)	*n*=20<br>0.85 (0.38–1.91) 0.99<br>± 0.45 (45%)	
HCV	*n*=7<br>1.06 (0.43–1.67) 1.07<br>± 0.37 (35%)	*n*=10<br>0.96 (0.58–5.39)<br>1.39 ± 1.43 (103%)	*n*=4<br>0.75 (0.04–1.69) 0.81<br>± 0.72 (88%)	*n*=21<br>0.99 (0.04–5.39) 1.17<br>± 1.04 (89%)	
PBC	*n*=2<br>1.14 (0.91–1.37) 1.14<br>± 0.33 (29%)	*n*=4<br>2.27 (0.63–4.61) 2.44<br>± 1.99 (81%)	*n*=4<br>0.63 (0.02–1.20) 0.62<br>± 0.55 (89%)	*n*=10<br>0.93 (0.02–4.61) 1.45<br>± 1.48 (102%)	
PSC	*n*=3<br>1.34 (0.96–2.34) 1.55<br>± 0.71 (46%)	*n*=3<br>0.97 (0.92–1.08) 0.99<br>± 0.08 (8%)	–	*n*=6<br>1.02 (0.92–2.34) 1.27<br>± 0.55 (43%)	
All diseases	*n*=18<br>1.29 (0.43–5.16) 1.96<br>± 1.43 (73%)	*n*=31<br>0.92 (0.38–5.39) 1.39<br>± 1.31 (94%)	*n*=28<br>0.93 (0.02–11.34)<br>1.41 ± 2.10 (149%)	*n*=77<br>0.97 (0.02–11.34)<br>1.53 ± 1.66 (109%)	
Controls					*n*=20<br>0.88 (0.33–2.81) 1.00<br>± 0.55 (55%)

number of samples (n); median values are given with range in brackets and mean values ± SD are provided in italics with CV% in brackets

Table S3. Protein abundance (fmol/mg tissue) of the P450s and UGTs in different liver pathologies (HCV—hepatitis C. PBC—primary biliary cholangitis. PSC—primary sclerosing cholangitis, ALD—alcoholic liver disease, AIH—autoimmune hepatitis) and disease stages (Child-Pugh class A, B and C) as well as in the controls.

	**Child-Pugh class**				
	**A**	**B**	**C**	**A+B+C**	**Controls**
			**CYP1A1**		
AIH	*n*=6<br>39.56 (0.00–114.71)<br>*45.32 ± 38.41 (85%)*<br>"5/6"	*n*=6<br>28.94 (0.00–80.33)<br>*32.20 ± 31.43 (98%)*<br>"4/6"	*n*=8<br>2.51 (0.00–53.61) *14.45*<br>*± 22.18 (153%)*<br>"4/8"	*n*=20<br>27.77 (0.00–114.71)<br>*29.04 ± 31.71 (109%)*<br>"13/20"	
ALD	–	*n*=8<br>10.52 (0.00–33.11)<br>*12.52 ± 9.33 (75%)*<br>"7/8"	*n*=12<br>13.49 (0.00–26.01)<br>*12.32 ± 8.75 (71%)*<br>"9/12"	*n*=20<br>12.10 (0.00–33.11)<br>*12.40 ± 8.74 (70%)*<br>"16/20"	
HCV	*n*=7<br>15.35 (0.00–26.18)<br>*15.34 ± 9.25 (60%)*<br>"6/7"	*n*=10<br>32.82 (12.53–76.00)<br>*36.57 ± 21.85 (60%)*<br>"10/10"	*n*=4<br>16.02 (11.06–95.98)<br>*34.77 ± 41.03 (118%)*<br>"4/4"	*n*=21<br>21.16 (0.00–95.98)<br>*29.15 ± 24.36 (84%)*<br>"20/21"	
PBC	*n*=2<br>5.76 (2.22–9.30)<br>*5.76 ± 5.01 (87%)*<br>"2/2"	*n*=4<br>0.00 (0.00–8.16)<br>*2.04 ± 4.08 (200%)*<br>"1/4"	*n*=4<br>7.73 (0.00–113.96)<br>*32.35 ± 54.89 (170%)*<br>"2/4"	*n*=10<br>1.11 (0.00–113.96)<br>*14.91 ± 35.22 (236%)*<br>"5/10"	
PSC	*n*=3<br>0.00 (0.00–9.92)	*n*=3<br>0.00	–	*n*=6<br>0.00 (0.00–9.92)	

	3.31 ± 5.73 (173%) "1/3"	0.00 "0/3"		*1.65 ± 4.05 (245%)* "1/6"	
All diseases	*n*=18 13.22 (0.00–114.71) *22.26 ± 27.79 (125%)* "14/18"	*n*=31 12.66 (0.00–80.33) *21.52 ± 23.38 (109%)* "22/31"	*n*=28 11.68 (0.00–113.96) *19.00 ± 27.74 (146%)* "19/28"	*n*=77 12.42 (0.00–114.71) *20.78 ± 25.77 (124%)* "55/77"	
Controls					*n*=20 21.50 (0.00–190.27) *37.68 ± 50.75 (135%)* "18/20"
			**CYP1A2**		
AIH	*n*=6 635.57 (217.89–1616.46) *802.57 ± 603.56 (75%)* "6/6"	*n*=6 1012.63 (106.30–1991.76) *965.32 ± 786.73 (81%)* "6/6"	*n*=8 183.33 (0.00–858.52) *321.23 ± 305.13 (95%)* "7/8"	*n*=20 536.35 (0.00–1991.76) *658.86 ± 614.18 (93%)* "19/20"	
ALD	–	*n*=8 173.49 (44.16–927.74) *266.22 ± 278.58 (105%)* "8/8"	*n*=12 175.52 (2.24–455.51) *189.77 ± 148.53 (78%)* "12/12"	*n*=20 175.18 (2.24–927.74) *220.35 ± 206.98 (94%)* "20/20"	
HCV	*n*=7 252.25 (123.73–486.67) *251.34 ± 126.91 (50%)* "7/7"	*n*=10 677.53 (95.53–2000.0) *861.63 ± 644.41 (75%)* "10/10"	*n*=4 104.35 (24.53–3019.78) *813.25 ± 1472.76 (181%)* "4/4"	*n*=21 318.30 (24.53–3019.78) *648.98 ± 774.86 (119%)* "21/21"	
PBC	*n*=2 387.19 (364.53–409.84) *387.19 ± 32.04 (8%)* "2/2"	*n*=4 265.22 (51.55–651.52) *308.38 ± 300.52 (97%)* "4/4"	*n*=4 116.25 (21.51–1509.80) *440.95 ± 717.82 (163%)* "4/4"	*n*=10 285.93 (21.51–1509.80) *377.17 ± 453.77 (120%)* "10/10"	
PSC	*n*=3 589.87 (499.15–1328.09) 805.70 ± 454.66 (56%) "3/3"	*n*=3 504.40 (203.18–1305.26) *670.95 ± 569.60 (85%)* "3/3"	–	*n*=6 547.14 (203.18–1328.09) *738.33 ± 466.81 (63%)* "6/6"	
All diseases	*n*=18 387.19 (123.73–1616.46) *542.57 ± 459.40 (85%)* "18/18"	*n*=31 443.38 (44.16–2000.00) *638.20 ± 605.03 (95%)* "31/31"	*n*=28 175.52 (0.00–3019.78) *352.28 ± 613.23 (174%)* "27/28"	*n*=77 268.98 (0.00–3019.78) *511.88 ± 584.31 (114%)* "76/77"	
Controls					*n*=20 768.45 (40.58–2645.59) *953.10 ± 738.45 (77%)* "20/20"
			**CYP2B6**		
AIH	*n*=6 77.49(33.40–144.03) *82.02 ± 37.69 (46%)* "6/6"	*n*=6 79.24(0.00–189.91) *85.97 ± 64.53 (75%)* "5/6"	*n*=8 42.11 (12.37–450.68) *93.28 ± 145.90(156%)* "8/8"	*n*=20 64.80 (0.00–450.68) *87.71 ± 96.62 (110%)* "19/20"	
ALD	_	*n*=8	*n*=12	*n*=20	

		47.58 (17.30–64.18)<br>*44.56 ± 16.69 (37%)*<br>"8/8"	60.70 (4.48–124.47)<br>*67.82 ± 36.38 (54%)*<br>"12/12"	49.42 (4.48–124.47)<br>*58.52 ± 31.71 (54%)*<br>"20/20"	
HCV	*n*=7<br>78.82 (42.98–121.67)<br>*82.94 ± 25.39 (31%)*<br>"7/7"	*n*=10<br>98.87 (44.39–327.99)<br>*114.06 ± 81.48 (71%)*<br>"10/10"	*n*=4<br>249.27 (36.82–253.54)<br>*197.22 ± 106.96(54%)*<br>"4/4"	*n*=21<br>89.35 (36.80–327.99)<br>*119.53 ± 81.16 (68%)*<br>"21/21"	
PBC	*n*=2<br>34.74 (29.97–39.51)<br>*34.74 ± 6.75 (19%)*<br>"2/2"	*n*=4<br>35.56 (0.00–45.16)<br>*29.07 ± 20.35 (70%)*<br>"3/4"	*n*=4<br>27.46 (0.00–213.84)<br>*67.19 ± 99.11 (148%)*<br>"3/4"	*n*=10<br>34.90 (0.00–213.84)<br>*45.45 ± 61.42 (135%)*<br>"8/10"	
PSC	*n*=3<br>104.54 (0.00–115.70)<br>*73.41 ± 63.82 (87%)*<br>"2/3"	*n*=3<br>136.83 (36.34–144.71)<br>*105.96 ± 60.42 (57%)*<br>"3/3"	–	*n*=6<br>110.12 (0.00–144.71)<br>*89.69 ± 58.37 (65%)*<br>"5/6"	
All diseases	*n*=18<br>75.91 (0.00–144.03)<br>*75.69 ± 36.90 (49%)*<br>"17/18"	*n*=31<br>62.36 (0.00–327.99)<br>*78.94 ± 64.62* (82%)<br>"29/31"	*n*=28<br>48.80 (0.00–450.68)<br>*93.49 ± 102.03(109%)*<br>"27/28"	*n*=77<br>64.18 (0.00–450.68)<br>*83.47 ± 75.57 (91%)*<br>"73/77"	
Controls					*n*=20<br>91.57 (14.28–292.68)<br>*103.05 ± 70.91 (69%)*<br>"20/20"
			**CYP2C8**		
AIH	*n*=6<br>470.87(252.35–579.86)<br>*441.26 ± 130.78 (30%)*<br>"6/6"	*n*=6<br>454.06(0.39–903.66)<br>*428.44 ± 309.33 (72%)*<br>"6/6"	*n*=8<br>141.39 (0.00–503.68)<br>*189.67 ± 165.44 (87%)*<br>"7/8"	*n*=20<br>335.60 (0.00–903.66)<br>*336.78 ± 234.47 (70%)*<br>"19/20"	
ALD	–	*n*=8<br>140.68 (58.78–198.71)<br>*144.21 ± 41.92 (29%)*<br>"8/8"	*n*=12<br>155.60 (8.97–376.41)<br>*188.30 ± 124.30 (66%)*<br>"12/12"	*n*=20<br>146.09 (8.97–376.41)<br>*170.67 ± 100.41 (59%)*<br>"20/20"	
HCV	*n*=7<br>234.13 (167.36–393.10)<br>*254.53 ± 87.77 (34%)*<br>"7/7"	*n*=10<br>296.40 (132.32–1282.94) *398.72 ± 334.70 (84%)*<br>"10/10"	*n*=4<br>456.50 (56.56–1061.03)<br>*507.65 ± 414.56 (82%)*<br>"4/4"	*n*=21<br>268.69 (56.56–1282.94)<br>*371.41 ± 295.58 (80%)*<br>"21/21"	
PBC	*n*=2<br>186.20 (143.85–228.55)<br>*186.20 ± 59.89 (32%)*<br>"2/2"	*n*=4<br>114.91 (48.94–178.37)<br>*114.28 ± 67.31 (59%)*<br>"4/4"	*n*=4<br>49.10 (9.51–572.53)<br>*170.06 ± 269.09 (158%)*<br>"4/4"	*n*=10<br>103.86 (9.51–572.53)<br>*150.98 ± 164.56 (109%)*<br>"10/10"	
PSC	*n*=3<br>590.39 (581.01–630.72)<br>*600.71 ± 26.41 (4%)*<br>"3/3"	*n*=3<br>545.43 (93.25–588.71)<br>*409.13 ± 274.42 (67%)*<br>"3/3"	–	*n*=6<br>584.86 (93.25–630.72)<br>*504.92 ± 203.50 (40%)*<br>"6/6"	
All diseases	*n*=18<br>335.76 (143.85–630.72)<br>*366.88 ± 169.36 (46%)*<br>"18/18"	*n*=31<br>198.71 (0.39–1282.94)<br>*303.10 ± 272.47 (90%)*<br>"31/31"	*n*=28<br>149.81 (0.00–1061.03)<br>*231.71 ± 231.81 (100%)*<br>"27/28"	*n*=77<br>215.32 (0.00–1282.94)<br>*292.05 ± 239.85 (82%)*<br>"76/77"	
Controls					*n*=20

					532.83 (101.21–1136.74) *538.74 ± 309.09 (57%)* “20/20”
			**CYP2C9**		
AIH	*n*=6 2333.27(1219.92–2912.02) *2196.38 ± 716.75 (33%)* “6/6”	*n*=6 2924.32(112.47–5308.10) *2632.59 ± 1808.96 (69%)* “6/6”	*n*=8 970.20 (68.76–2828.47) *1212.08 ± 853.85 (70%)* “8/8”	*n*=20 1722.15 (68.76–5308.10) *1933.52 ± 1288.50 (67%)* “20/20”	
ALD	–	*n*=8 1216.58 (499.33–1820.94) *1220.81 ± 458.02(38%)* “8/8”	*n*=12 1419.25 (0.00–3040.05) *1325.52 ± 929.52 (70%)* “11/12”	*n*=20 1288.66 (0.00–3040.05) *1283.64 ± 761.76 (59%)* “19/20”	
HCV	*n*=7 1545.98 (686.34–2582.00) *1623.71 ± 566.48 (35%)* “7/7”	*n*=10 1831.92 (463.05–5358.50) *1998.47 ± 1354.68 (68%)* “10/10”	*n*=4 1369.05 (511.79–3599.48) *1712.34 ± 1329.46 (78%)* “4/4”	*n*=21 1636.92 (463.05–5358.50) *1819.05 ± 1104.06 (61%)* “21/21”	
PBC	*n*=2 1718.56 (1415.27–2021.85) *1718.56 ± 428.91 (25%)* “2/2”	*n*=4 1571.10 (915.69–2358.76) *1604.16 ± 606.85 (38%)* “4/4”	*n*=4 675.22 (411.46–5709.46) *1867.89 ± 2565.94 (137%)* “4/4”	*n*=10 1407.16 (411.46–5709.67) *1732.53 ± 1534.08 (89%)* “10/10”	
PSC	*n*=3 2968.45 (2683.52–4384.83) *3345.60 ± 977.21 (27%)* “3/3”	*n*=3 3046.67 (1046.60–3085.78) *2393.02 ± 1166.19 (49%)* “3/3”	–	*n*=6 3007.56 (1046.60–4384.83) *2869.31 ± 1071.61 (37%)* “6/6”	
All diseases	*n*=18 1971.59 (686.34–4384.83) *2112.12 ± 871.96 (41%)* “18/18”	*n*=31 1799.80 (112.47–5358.50) *1907.82 ± 1241.72 (65%)* “31/31”	*n*=28 1130.30 (0.00–5709.67) *1425.85 ± 1236.16 (87%)* “27/28”	*n*=77 1548.11 (0.00–5709.67) *1780.32 ± 1183.46 (66%)* “76/77”	
Controls					*n*=20 2007.57 (630.81–5676.82) *2290.74 ± 1276.02 (56%)* “20/20”
			**CYP2C19**		
AIH	*n*=6 342.38(189.33–497.87) *351.50 ± 127.20 (36%)* “6/6”	*n*=6 214.20(105.35–1378.41) *400.19 ± 489.90 (122%)* “6/6”	*n*=8 284.81 (0.00–1068.23) *382.69 ± 323.89 (85%)* “7/8”	*n*=20 248.81 (0.00–1378.41) *378.58 ± 326.27 (86%)* “19/20”	
ALD		*n*=8 166.36 (62.62–356.60) *175.08 ± 92.99 (53%)* 8/8	*n*=12 274.20 (0.00–671.56) *293.80 ± 200.88 (68%)* 11/12	*n*=20 215.57 (0.00–671.56) *246.31 ± 173.52 (70%)* “19/20”	

HCV	*n*=7 238.04 (151.13–379.91) *251.85 ± 72.43 (29%)* "7/7"	*n*=10 202.01 (0.00–782.00) *247.66 ± 206.22 (83%)* "9/10"	*n*=4 260.27 (175.14–1067.24) *440.73 ± 419.83 (95%)* "4/4"	*n*=21 226.92 (0.00–1067.24) *285.83 ± 230.39 (81%)* "20/21"	
PBC	*n*=2 212.34 (111.77–312.91) *212.34 ± 142.22 (67%)* "2/2"	*n*=4 283.92 (220.22–314.62) *275.67 ± 42.41 (15%)* "4/4"	*n*=4 556.94 (170.54–1520.93) *701.33 ± 638.86 (91%)* "4/4"	*n*=10 283.92 (111.77–1520.93) *433.27 ± 438.99 (101%)* "10/10"	
PSC	*n*=3 269.74 (255.35–348.88) *291.32 ± 50.37 (17%)* "3/3"	*n*=3 584.17 (426.61–696.24) *569.01 ± 135.45 (24%)* "3/3"	–	*n*=6 387.75 (255.35-696.24) *430.17 ± 177.44 (41%)* "6/6"	
All diseases	*n*=18 263.36 (111.77-497.87) *287.26 ± 103.61 (36%)* "18/18"	*n*=31 222.97 (0.00-1378.41) *293.16 ± 265.57 (91%)* "30/31"	*n*=28 278.15 (0.00-1520.93) *398.41 ± 356.88 (90%)* "26/28"	*n*=77 255.35 (0.00-1520.93) *330.05 ± 279.64 (85%)* "74/77"	
Controls					*n*=18 332.11 (81.13–797.67) *373.86 ± 182.53 (49%)* "18/18"
**CYP2D6**					
AIH	*n*=6 403.69(93.06–1655.28) *562.77 ± 552.41 (98%)* "6/6"	*n*=5 440,01(24,03–655.32) *384.11 ± 230.44 (60%)* "5/5"	*n*=8 284.27 (174.65–579.32) *334.12 ± 142.02 (43%)* "8/8"	*n*=19 363.99 (24.03–1655.28) *419.48 ± 338.88 (81%)* "19/19"	
ALD	–	*n*=8 140.65 (43.95–270.36) *145.65 ± 83.49(57%)* "8/8"	*n*=12 168.46 (0.00–309.62) *151.68 ± 95.68 (63%)* "10/12"	*n*=20 161.89 (0.00–309.62) *49.27 ± 88.75 (59%)* "18/20"	
HCV	*n*=7 187.77 (119.08–373.72) *219.03 ± 90.43 (41%)* "7/7"	*n*=8 395.26 (148.75–987.34) *415.31 ± 265.58 (64%)* "8/8"	*n*=4 165.89 (138.96–507.12) *244.46 ± 175.57 (72%)* "4/4"	*n*=19 244.65 (119.08–987.34) *307.03 ± 210.68 (69%)* "19/19"	
PBC	*n*=2 207.09 (196.81–217.36) *207.09 ± 14.54 (7%)* "2/2"	*n*=4 321.63 (145.73–594.24) *345.81 ± 185.55 (54%)* "4/4"	*n*=3 390.65 (216.97–1414.72) *674.11 ± 647.24 (96%)* "3/3"	*n*=9 308.15 (145.73–1414.72) *424.42 ± 394.90 (93%)* "9/9"	
PSC	*n*=3 641.72 (21.82–836.80) *500.11 ± 425.55 (85%)* "3/3"	*n*=3 483.19 (283.91–828.98) *532.03 ± 275.80 (52%)* "3/3"	–	*n*=6 562.46 (21.82–836.80) *516.07 ± 321.20 (62%)* "6/6"	
All diseases	*n*=18 269.93 (21.82–1655.28) *379.13 ± 377.46 (100%)*	*n*=28	*n*=27	*n*=73 253.89 (0.00–1655.28) *324.73 ± 285.79 (88%)*	

	"18/18"	296.03 (24.03–987.34)<br>*335.27 ± 233.95 (70%)*<br>"28/28"	211.82 (0.00–1414.72)<br>*277.53 ± 267.76 (96%)*<br>"25/27"	"71/73"	
Controls					*n*=19<br>292.45 (55.71–852.82) *346.67 ± 239.57 (66%)*<br>"19/19"
**CYP2E1**					
AIH	*n*=6<br>1001.71(644.88–1551.35) *1033.48 ± 293.54 (28%)*<br>"6/6"	*n*=6<br>889.09(121.03–2274.78) *1156.33 ± 867.84 (75%)*<br>"6/6"	*n*=8<br>770.02 (56.54–1473.93)<br>*710.34 ± 495.11 (70%)*<br>"8/8"	*n*=20<br>918.66 (56.54–2274.78)<br>*941.08 ± 592.39 (63%)*<br>"20/20"	
ALD	–	*n*=8<br>457.81 (86.24–1592.72) *518.64 ± 467.60 (90%)*<br>"8/8"	*n*=12<br>510.58 (6.72–1101.62)<br>*556.74 ± 339.48 (61%)*<br>"12/12"	*n*=20<br>466.94 (6.72–1592.72)<br>*541.50 ± 384.24 (71%)*<br>"20/20"	
HCV	*n*=7<br>504.42 (291.22–800.64)<br>*531.95 ± 175.61 (33%)*<br>"7/7"	*n*=10<br>695.04 (401.48–3413.52)<br>*964.74 ± 892.22 (92%)*<br>"10/10"	*n*=4<br>940.92 (402.76–1304.53) *897.28 ± 442.76 (49%)*<br>"4/4"	*n*=21<br>646.04 (291.22–3413.52) *807.63 ± 661.39 (82%)*<br>"21/21"	
PBC	*n*=2<br>597.60 (549.34–645.86)<br>*597.60 ± 68.25 (11%)*<br>"2/2"	*n*=4<br>613.54 (413.80–766.95) *601.96 ± 171.01 (28%)*<br>"4/4"	*n*=4<br>471.60 (288.06–1225.31) *614.14 ± 424.13 (69%)*<br>"4/4"	*n*=10<br>559.42 (288.06–1225.31) *605.96 ± 265.10 (44%)*<br>"10/10"	
PSC	*n*=3<br>913.02 (576.46–1057.64) *849.04 ± 246.89 (29%)*<br>"3/3"	*n*=3<br>324.89 (301.85–1616.24) *747.66 ± 752.30 (101%)*<br>"3/3"	–	*n*=6<br>744.74 (301.85–1616.24) *798.35 ± 503.83 (63%)*<br>"6/6"	
All diseases	*n*=18<br>684.40 (291.22–1551.35) *759.27 ± 309.81 (41%)*<br>"18/18"	*n*=31<br>622.97 (86.24–3413.52) *818.88 ± 718.82 (88%)*<br>"31/31"	*n*=28<br>607.77 (6.72–1473.93)<br>*657.47 ± 407.94 (62%)*<br>"28/28"	*n*=77<br>644.88 (6.72–3413.52)<br>*746.25 ± 538.19 (72%)*<br>"77/77"	
Controls					*n*=20<br>1394.06 (272.07–3407.44) *1549.54 ± 814.53 (53%)*<br>"20/20"
**CYP3A4**					
AIH	*n*=6<br>470.82(176.76–768.15)<br>*464.15 ± 210.63 (45%)*<br>"6/6"	*n*=6<br>597.91(0.00–1174.48)<br>*574.65 ± 417.30 (73%)*<br>"5/6"	*n*=8<br>205.83 (0.00–871.11)<br>*251.98 ± 294.59 (117%)*<br>"5/8"	*n*=20<br>366.52 (0.00–1174.48)<br>*412.43 ± 330.86 (80%)*<br>"16/20"	
ALD	–	*n*=8	*n*=12	*n*=20	

		217.91 (67.05–626.39) *257.26 ± 180.36 (70%)* “8/8″	245.05 (0.00–963.87) *263.61 ± 268.57 (102%)* “10/12″	220.25 (0.00–963.87) *261.07 ± 231.85 (89%)* “18/20″	
HCV	*n*=7 323.59 (243.61–736.27) *403.92 ± 190.53 (47%)* “7/7″	*n*=10 270.79 (114.46–1126.37) *373.43 ± 303.73 (81%)* “10/10″	*n*=4 686.36 (0.00–1421.24) *698.49 ± 764.08 (109%)* “3/4″	*n*=21 305.34 (0.00–1421.24) *445.51 ± 394.94 (89%)* “20/21″	
PBC	*n*=2 810.48 (459.10–1161.86) *810.48 ± 496.93 (61%)* “2/2″	*n*=4 167.63 (0.00–721.38) *264.16 ± 318.74 (121%)* “3/4″	*n*=4 15.88 (0.00–433.16) *116.23 ± 211.82 (182%)* “2/4″	*n*=10 167.63 (0.00–1161.86) *314.25 ± 386.68 (123%)* “7/10″	
PSC	*n*=3 393.30 (241.31–2219.47) 591.36 ± 1100.84 *(116%)* “3/3″	*n*=3 517.25 (162.44–9025.86) *3235.18 ± 5018.01 (155%)* “3/3″	–	*n*=6 455.28 (162.44–9025.86) *2093.27 ± 3481.62 (166%)* “6/6″	
All diseases	*n*=18 402.72 (176.76–2219.47) *560.41 ± 480.25 (86%)* “18/18″	*n*=31 255.46 (0.00–9025.86) *645.24 ± 1584.00 (245%)* “29/31″	*n*=28 182.98 (0.00–1421.24) *301.36 ± 389.28 (129%)* “20/28″	*n*=77 299.87 (0.00–9025.86) *500.36 ± 1058.23 (211%)* “67/77″	
Controls					*n*=20 672.99 (99.21–2634.15) *967.18 ± 745.25 (77%)* “20/20″
			**UGT1A1**		
AIH	*n*=6 1315.57(658.66–1769.78) *1294.39 ± 471.88 (36%)* “6/6″	*n*=6 626.25(208.99–2743.40) *1099.93 ± 1092.17 (99%)* “6/6″	*n*=8 643.96 (170.44–1363.23) *733.37 ± 428.18 (58%)* “8/8″	*n*=20 780.88 (170.44–2743.40) *1011.64 ± 707.36 (70%)* “20/20″	
ALD	–	*n*=8 394.69 (30.49–945.65) *454.00 ± 344.00 (76%)* “8/8″	*n*=12 649.29 (0.00–1332.91) *682.57 ± 401.33 (59%)* “11/12″	*n*=20 542.55 (0.00–1332.91) *591.14 ± 387.35 (66%)* “19/20″	
HCV	*n*=7 500.57 (390.06–928.94) *609.75 ± 236.40 (39%)* “7/7″	*n*=10 1029.00 (95.74–1648.75) *924.58 ± 484.80 (52%)* “10/10″	*n*=4 901.69 (496.80–1860.83) *1040.25 ± 588.99 (57%)* “4/4″	*n*=21 795.95 (95.74–1860.83) *841.67 ± 452.45 (54%)* “21/21″	
PBC	*n*=2 701.66 (516.30–887.01) *701.66 ± 262.14 (37%)* “2/2″	*n*=4 771.09 (554.58–903.81) *750.14 ± 173.99 (23%)* “4/4″	*n*=4 441.49 (176.97–3086.67) *1036.66 ± 1374.11 (133%)* “4/4″	*n*=10 603.59 (176.97–3086.67) *855.05 ± 819.69 (96%)* “10/10″	
PSC	*n*=3	*n*=3	–	*n*=6	

	1143.77 (706.59–1526.54) *1125.64 ± 410.28 (36%)* "3/3"	1436.95 (898.12–1598.19) *1311.08 ± 366.61 (28%)* "3/3"		1290.36 (706.59–1598.19) *1218.36 ± 362.51 (30%)* "6/6"	
All diseases	*n*=18 896.47 (390.06–1769.78) *934.16 ± 459.37 (49%)* "18/18"	*n*=31 803.90 (30.49–2743.40) *851.98 ± 622.62 (73%)* "31/31"	*n*=28 649.29 (0.00–3086.67) *798.77 ± 621.11 (78%)* "27/28"	*n*=77 757.87 (0.00–3086.67) *851.84 ± 583.03 (68%)* "76/77"	
Controls					*n*=20 619.52 (182.88–1123.72) *641.33 ± 309.24 (48%)* "20/20"
			**UGT1A3**		
AIH	*n*=6 289.58(190.72–748.24) *358.79 ± 210.87 (59%)* "6/6"	*n*=6 347.67(220.34–470.40) *354.73 ± 91.42 (26%)* "6/6"	*n*=8 310.96 (114.04–855.38) *348.40 ± 233.39 (67%)* "8/8"	*n*=20 337.14 (114.04–855.38) *353.42 ± 184.36 (52%)* "20/20"	
ALD	–	*n*=8 76.16 (32.13–157.78) *79.16 ± 37.92 (48%)* "8/8"	*n*=12 119.56 (45.06–308.11) *131.35 ± 81.00 (62%)* "12/12"	*n*=20 90.83 (32.13–308.11) *110.47 ± 70.82 (64%)* "20/20"	
HCV	*n*=7 74.18 (22.14–295.96) *103.74 ± 102.50 (99%)* "7/7"	*n*=10 137.24 (30.98–358.13) *152.64 ± 102.42 (67%)* "10/10"	*n*=4 120.88 (73.17–329.48) *161.10 ± 120.55 (75%)* "4/4"	*n*=21 100.44 (22.14–358.13) *137.95 ± 103.33 (75%)* "21/21"	
PBC	*n*=2 265.37 (245.46–285.29) *265.37 ± 28.16 (11%)* "2/2"	*n*=4 362.23 (70.14–728.09) *380.67 ± 300.10 (79%)* "4/4"	*n*=4 332.97 (67.81–503.06) *309.21 ± 195.50 (63%)* "4/4"	*n*=10 265.37 (67.81–728.09) *329.03 ± 212.39 (65%)* "10/10"	
PSC	*n*=3 360.24 (159.33–505.24) *341.60 ± 173.70 (51%)* "3/3"	*n*=3 484.78 (193.10–820.17) *499.35 ± 313.79 (63%)* "3/3"	–	*n*=6 422.51 (159.33–820.17) *420.48 ± 242.73 (58%)* "6/6"	
All diseases	*n*=18 220.15 (22.14–748.24) *245.36 ± 186.71 (76%)* "18/18"	*n*=31 163.55 (30.98–820.17) *235.77 ± 204.62 (87%)* "31/31"	*n*=28 162.98 (45.06–855.38) *223.02 ± 180.88 (81%)* "28/28"	*n*=77 190.15 (22.14–855.38) *233.61 ± 189.82 (81%)* "77/77"	
Controls					*n*=20 205.20 (80.90–657.50) *235.37 ± 151.87 (65%)* "20/20"
			**UGT2B7**		
AIH	*n*=6 1679.76(862.77–3127.55) *1833.02 ± 786.00 (43%)*	*n*=6 1994.21(772.47–3727.61) *1997.53 ± 1042.32 (52%)*	*n*=8 604.69 (52.62–2256.12) *871.19 ± 734.30 (84%)* "8/8"	*n*=20 1471.71 (52.62–3727.61) *1497.64 ± 962.71 (64%)*	

	"6/6"	"6/6"		"20/20"	
ALD	–	*n*=8<br>690.45 (0.00–993.91)<br>*650.81 ± 288.59 (44%)*<br>"7/8"	*n*=12<br>876.24 (0.00–2948.18)<br>*1053.98 ± 784.39 (74%)*<br>"11/12"	*n*=20<br>722.60 (0.00–2948.18)<br>*892.71 ± 654.18 (73%)*<br>"18/20"	
HCV	*n*=7<br>965.07 (725.53–1840.67) *1130.82 ± 449.77 (40%)*<br>"7/7"	*n*=10<br>912.47 (0.00–3642.30)<br>*1057.43 ± 971.65 (92%)*<br>"9/10"	*n*=4<br>331.06 (0.00–424.27)<br>*271.60 ± 194.10 (71%)*<br>"3/4"	*n*=21<br>792.63 (0.00–3642.30)<br>*932.21 ± 774.67 (83%)*<br>"19/21"	
PBC	*n*=2<br>1136.28 (1109.80–1162.76) *1136.28 ± 37.44 (3%)*<br>"2/2"	*n*=4<br>942.25 (511.68–1344.34) *935.13 ± 342.57 (37%)*<br>"4/4"	*n*=4<br>361.84 (166.53–3418.49) *1077.18 ± 1565.71 (145%)* "4/4"	*n*=10<br>404.27 (86.41–1994.89)<br>*491.58 ± 548.61 (112%)*<br>"10/10"	
PSC	*n*=3<br>2240.39 (1410.85–3263.92) *2305.05 ± 928.23 (40%)*<br>"3/3"	*n*=3<br>1433.41 (458.79–2860.46) *1584.22 ± 1207.91 (76%)*<br>"3/3"	–	*n*=6<br>1836.90 (458.79–3263.92) *1944.63 ± 1041.22 (54%)*<br>"6/6"	
All diseases	*n*=18<br>1384.25 (725.53–3263.92) *1561.20 ± 758.80 (49%)*<br>"18/18"	*n*=31<br>891.30 (0.00–3727.61)<br>*1169.65 ± 911.22 (78%)*<br>"29/31"	*n*=28<br>596.73 (0.00–3418.49)<br>*893.30 ± 860.76 (96%)*<br>"26/28"	*n*=77<br>891.30 (0.00–3727.61)<br>*1160.69 ± 885.51 (76%)*<br>"73/77"	
Controls					*n*=20<br>1700.99 (344.22–3455.79) *1904.59 ± 913.12 (48%)*<br>"20/20"
**UGT2B15**					
AIH	*n*=6<br>592.57 (296.81–800.97)<br>*581.58 ± 186.47 (32%)*<br>"6/6"	*n*=6<br>670.81 (345.64–1109.12) *690.56 ± 331.90 (48%)*<br>"6/6"	*n*=8<br>362.03 (24.57–1343.02)<br>*445.81 ± 397.58 (89%)*<br>"8/8"	*n*=20<br>479.25 (24.57–1343.02)<br>*559.96 ± 327.71 (59%)*<br>"20/20"	
ALD	–	*n*=8<br>322.42 (143.92–432.71) *323.47 ± 90.18 (28%)*<br>"8/8"	*n*=12<br>409.34 (0.00–1296.64)<br>*458.89 ± 345.74 (75%)*<br>"11/12"	*n*=20<br>359.41 (0.00–1296.64)<br>*404.72 ± 277.19 (68%)*<br>"19/20"	
HCV	*n*=7<br>405.64 (157.13–670.11)<br>*434.20 ± 190.91 (44%)*<br>"7/7"	*n*=10<br>357.36 (221.01–1637.92) *495.61 ± 435.03 (88%)*<br>"10/10"	*n*=4<br>297.26 (101.54–2245.26) *735.33 ± 1012.89 (138%)*<br>"4/4"	*n*=21<br>367.60 (101.54–2245.26) *520.80 ± 511.99 (98%)*<br>"21/21"	
PBC	*n*=2<br>467.37 (464.46–470.27)<br>*467.37 ± 4.11 (1%)*<br>"2/2"	*n*=4<br>404.27 (272.97–482.67) *391.04 ± 89.30 (23%)*<br>"4/4"	*n*=4<br>167.80 (86.41–1994.89)<br>*604.22 ± 929.59 (154%)*<br>"4/4"	*n*=10<br>404.27 (86.41–1994.89)<br>*491.58 ± 548.61 (112%)*<br>"10/10"	

PSC	*n*=3<br>847.94 (619.48–1324.00) *930.47 ± 359.44 (39%)*<br>"3/3"	*n*=3<br>733.30 (331.31–870.14) *644.92 ± 280.08 (43%)*<br>"3/3"	–	*n*=6<br>790.62 (331.31–1324.00) *787.69 ± 327.90 (42%)*<br>"6/6"	
All diseases	*n*=18<br>553.30 (157.13–1324.00) *569.72 ± 264.77 (46%)*<br>"18/18"	*n*=31<br>379.41 (143.92–1637.92) *489.88 ± 319.71 (65%)*<br>"31/31"	*n*=28<br>368.80 (0.00–2245.26)<br>*515.40 ± 557.56 (108%)*<br>"27/28"	*n*=77<br>405.00 (0.00–2245.26)<br>*517.82 ± 409.18 (79%)*<br>"76/77"	
Controls					*n*=20<br>550.30 (174.36–1670.44) *629.15 ± 346.94 (55%)*<br>"20/20"

number of samples (n); median values are given with range in brackets and mean values ± SD are provided in italics with CV% in brackets; positive samples: number of samples with detectable level of the protein in quotes

Table S4. Differences in the drug metabolizing enzymes protein abundance and mRNA levels between studied groups (the Kruskal-Wallis test followed by Dunn's multiple comparison test with Bonferroni correction). The p values are shown in the table. Bold font indicates p value <0.05.

**mRNA level**	***CYP1A1***	***CYP1A2***	***CYP2B6***	***CYP2C8***	***CYP2C9***	***CYP2C19***	***CYP2D6***	***CYP2E1***	***CYP3A4***	***UGT1A1***	***UGT1A3***	***UGT2B7***	***UGT2B15***
						***p* value**							
AIH vs ALD	1.000	1.000	0.100	1.000	0.042	1.000	1.000	0.969	0.513	1.000	1.000	1.000	1.000
AIH vs HCV	1.000	1.000	0.000	1.000	1.000	1.000	1.000	1.000	1.000	1.000	1.000	1.000	1.000
AIH vs PBC	1.000	1.000	1.000	1.000	0.155	1.000	1.000	1.000	1.000	1.000	1.000	1.000	1.000
AIH vs PSC	1.000	1.000	0.542	1.000	1.000	0.378	1.000	1.000	1.000	1.000	1.000	1.000	1.000
ALD vs HCV	0.920	0.952	0.543	0.088	0.023	1.000	1.000	0.167	0.625	1.000	1.000	0.704	1.000
ALD vs PBC	1.000	1.000	0.713	1.000	1.000	1.000	1.000	1.000	1.000	1.000	1.000	1.000	1.000
ALD vs PSC	1.000	1.000	1.000	1.000	1.000	0.114	1.000	1.000	0.019	1.000	1.000	1.000	1.000
HCV vs PBC	1.000	1.000	0.003	0.033	0.105	1.000	1.000	1.000	1.000	1.000	1.000	0.762	1.000
HCV vs PSC	1.000	1.000	1.000	1.000	1.000	0.834	1.000	1.000	0.935	1.000	1.000	1.000	1.000
PBC vs PSC	1.000	1.000	1.000	0.406	1.000	0.577	1.000	1.000	0.114	1.000	1.000	1.000	1.000
Ch-P A vs B	1.000	1.000	1.000	1.000	0.753	0.264	0.101	1.000	1.000	1.000	1.000	0.880	0.173
Ch-P A vs C	1.000	0.187	1.000	0.186	0.004	0.002	0.016	0.260	0.010	1.000	1.000	0.006	0.147
Ch-P B vs C	1.000	0.216	1.000	1.000	0.177	0.345	1.000	0.159	0.110	1.000	1.000	0.190	1.000
**Protein abundance [fmol/mg tissue]**	**CYP1A1**	**CYP1A2**	**CYP2B6**	**CYP2C8**	**CYP2C9**	**CYP2C19**	**CYP2D6**	**CYP2E1**	**CYP3A4**	**UGT1A1**	**UGT1A3**	**UGT2B7**	**UGT2B15**
						***p* value**							
AIH vs ALD	1.000	0.164	1.000	0.305	1.000	1.000	0.001	0.218	1.000	0.683	0.000	0.513	1.000

AIH vs HCV	1.000	1.000	0.795	1.000	1.000	1.000	1.000	1.000	1.000	1.000	0.000	0.671	1.000
AIH vs PBC	0.186	1.000	0.563	0.348	1.000	1.000	1.000	1.000	1.000	1.000	1.000	1.000	1.000
AIH vs PSC	0.080	1.000	1.000	1.000	1.000	1.000	1.000	1.000	1.000	1.000	1.000	1.000	1.000
ALD vs HCV	0.402	0.485	0.132	0.110	1.000	1.000	0.149	1.000	1.000	1.000	1.000	1.000	1.000
ALD vs PBC	1.000	1.000	1.000	1.000	1.000	1.000	0.070	1.000	1.000	1.000	0.013	1.000	1.000
ALD vs PSC	0.797	0.144	1.000	0.021	0.040	0.300	0.013	1.000	0.652	0.065	0.004	0.290	0.168
HCV vs PBC	0.015	1.000	0.004	0.157	1.000	1.000	1.000	1.000	1.000	1.000	0.072	1.000	1.000
HCV vs PSC	0.009	1.000	1.000	1.000	0.752	0.413	1.000	1.000	1.000	1.000	0.021	0.357	0.306
PBC vs PSC	1.000	1.000	0.487	0.023	0.341	1.000	1.000	1.000	1.000	0.503	1.000	0.934	0.435
Ch-P A vs B	1.000	1.000	1.000	0.476	1.000	1.000	1.000	1.000	0.770	1.000	1.000	0.280	0.785
Ch-P A vs C	1.000	0.090	1.000	0.031	0.047	1.000	1.000	1.000	0.032	1.000	1.000	0.006	0.224
Ch-P B vs C	1.000	0.051	1.000	1.000	0.350	0.660	1.000	1.000	0.793	1.000	1.000	0.754	1.000

AIH - autoimmune hepatitis, ALD—alcoholic liver disease, HCV—hepatitis C, PBC—primary biliary cholangitis, PSC—primary sclerosing cholangitis, Ch-P A, B, C- Child Pugh class A, B, C
